# Malleability of rumination: An exploratory model of CBT-based plasticity and long-term reduced risk for depressive relapse among youth from a pilot randomized clinical trial

**DOI:** 10.1371/journal.pone.0233539

**Published:** 2020-06-17

**Authors:** Katie L. Bessette, Rachel H. Jacobs, Charlotte Heleniak, Amy T. Peters, Robert C. Welsh, Edward R. Watkins, Scott A. Langenecker

**Affiliations:** 1 Department of Psychiatry, University of Illinois at Chicago, Chicago, IL, United States of America; 2 Department of Psychiatry, University of Utah, Salt Lake City, UT, United States of America; 3 Department of Psychiatry and Behavioral Sciences, Northwestern University Feinberg School of Medicine, Chicago, IL, United States of America; 4 Department of Psychology, Columbia University, New York City, NY, United States of America; 5 Department of Psychiatry, Massachusetts General Hospital, Boston, MA, United States of America; 6 Department of Psychiatry, Harvard Medical School, Boston, MA, United States of America; 7 Mood Disorders Centre, University of Exeter, Exeter, United Kingdom; Cornelius Vanderbilt Professor of Psychology & Human Development, UNITED STATES

## Abstract

Early-onset depression is associated with increased lifespan chronicity, suicidality, and the functional burdens associated with recurrent episodes. Reduction of these negative long-term outcomes requires an understanding of what interventions reduce vulnerabilities or ameliorate their associated neural patterns. Rumination-Focused Cognitive Behavior Therapy (RFCBT), an evidence-based treatment which directly targets a well-known risk for depressive relapse (i.e., ruminative habit), has shown preliminary success for reducing relapse rates among adolescents and adults in remission from Major Depressive Disorder (rMDD). In the current pilot study, clinical outcomes and neuroimaging predictors are explored over two years following a randomized controlled trial of eight weeks of RFCBT (*n* = 17) or Assessment Only (AO; *n* = 16) to prevent depressive relapse among adolescents. Baseline neural activation during a rumination induction task, neural change during treatment, and treatment group are examined as predictors of relapse. Regions selected were based upon hyperactivation patterns in rMDD compared to healthy adolescents, and occurred largely in default mode, somatomotor, visual and salience network regions. Twenty-five adolescents successfully completed quality fMRI scans at Baseline and after eight weeks of RFCBT or AO. RFCBT-treated youth maintained lower depression scores over the two-year period, relapsed at slower and less frequent rates, and were hospitalized less often for suicidality than AO. Although both groups reported lower ruminative tendencies after one year, at two-year follow-up, RFCBT maintained reductions whereas AO reported a return of ruminative tendencies. Adolescents who demonstrated higher activation at Baseline in default mode and limbic regions during rumination induction reported reduced depressive symptoms over the follow-up period. These data offer preliminary evidence that targeting rumination can stave off depressive relapse among vulnerable adolescents, and that such targeted treatment may aid a compensation process for disease-related brain functioning reducing risk to further depressive episodes.

**Clinical trials registration:**

NCT01905267, https://clinicaltrials.gov/ct2/show/NCT01905267

## Introduction

Twenty-five percent of individuals with Major Depressive Disorder (MDD) experience their first episode before age 19 [1]. Adolescent-onset MDD is associated with more chronic, severe, and recurrent episodes and greater functional impairment (e.g., educational and occupational underachievement) [2–5], contributing to a main cause of adult disability and reduced life quality across the lifespan [6, 7]. In addition, adolescents who recover from a depressive episode remain at elevated risk for relapse [8]. Few studies have examined treatments for the prevention of relapse or recurrence, despite these youths’ high risk. Even more concerning is the growing evidence for a cumulative neurobiological burden of repeated episodes of depression [9, 10] and the potential for worse outcomes if treatment is delayed [11]. To increase efficacy of relapse prevention services for youth, we must fully explicate the specific behavioral and brain mechanisms contributing to depressive resilience and relapse.

Rumination is a well-known risk factor for depressive relapse among adolescents and adults [9, 12]. Rumination is a habitual mental response to difficulties and associated negative emotions with a repetitive, passive, and abstract mental pattern or habit, in contrast to a more adaptive concrete and specific mental pattern (e.g., problem-solving, emotional processing) [13, 14]. The neural correlates of rumination are typically identified within the subgenual prefrontal cortex and key nodes of the default mode network (DMN), including the inferior parietal cortex (IPC), medial prefrontal cortex (mPFC) and posterior cingulate cortex (PCC) [15–17]. The DMN is hypothesized to support a broad array of mental processes, including mind-wandering, self-referential processing, and self-generated thought in both healthy and disease-related functioning [18]. Both resting state and mood-induction functional MRI tasks, such as self-referential word categorization, have implicated elevated activation and connectivity between DMN nodes as supporting increased ruminative tendencies [16, 19–23], although see [24]. These authors also found that increased rumination correlated with activity and connectivity in visual and somatosensory cortices. Given that common consequences of rumination include exacerbated negative mood (e.g.,[25]), it is not surprising that activity in and connectivity with regions of the Salience and Emotion Network (SEN), such as the amygdala, are also observed in response to rumination induction tasks [21, 26, 27].

It is currently unclear whether findings of the DMN, visual, limbic, and somatosensory regions are directly attributable to rumination as opposed to concurrent depressive symptoms. In numerous tasks, activation and connectivity between DMN nodes has also been associated with depressive symptomatology, both in those with current depression, and in those with mild subthreshold symptoms [16, 28]. For example, depressed adults exhibited greater activation in the PCC, mPFC, and parahippocampus (PHG) when rumination was induced through task demands [26]. Additionally, depressed youth exhibited greater connectivity within the DMN and between the DMN and cingulate and striatum (SEN) during both resting-state and emotion identification tasks [29].

Changes in ruminative habits and depressive symptoms appear to have simultaneous effects in DMN activity. In adults, reductions in depressive symptoms contemporaneous with changes in self-referential habits via prescribed medications was associated with reductions in abnormally elevated mPFC activity [30]. Moreover, psychotherapy with depressed adults also illustrated concurrent reductions in abnormal hyperactivation of mPFC during self-referential processing of negative words [31].

Importantly, individuals in remission from depression continue to exhibit higher ruminative tendencies than their healthy counterparts (e.g., [32]). Studies examining DMN connectivity in relation to ruminative tendencies among remitted adolescents and adults have been mixed, leading to uncertainty regarding whether such hyperconnectivity is a disease marker or malleable treatment target. Although several studies have found DMN hyperconnectivity in remission related to depressive symptoms and ruminative tendencies [33, 34], one study found stable and test-retest reliable hypoconnectivity specifically in ventral DMN regions was related to depressive symptoms and ruminative tendencies [35]. Such mixed findings make it difficult to determine whether DMN connectivity is a disease marker or malleable intervention target. Such complexities in the literature may become clearer using task-based activation patterns during rumination induction, rather than correlating self-reported rumination with connectivity.

We previously reported cross-sectional results from the current sample, comparing adolescents in remission from depression (rMDD) to adolescents free of psychiatric history on neural correlates of a rumination induction task [36]. rMDD adolescents demonstrated greater activation during rumination (versus distraction) in regions encompassing the DMN (including left precuneus and right IPL), visual and somatosensory regions, limbic regions (including amygdala, thalamus, and insula), as well as left MTG and left fusiform gyrus (S1 Fig and S1 Table). These group differences in rumination-associated activation were represented by two latent factors, determined via exploratory factor analysis on extracted z-values of all significant clusters. The first factor primarily consisted of salience-emotion, visual and somatomotor regions (hereafter referred to as SV-SM), whereas the second factor consisted of posterior default mode, limbic, and visual regions (hereafter referred to as pDMN+). Notably, both factors were positively correlated with self-reported rumination and clinician-reported depressive symptoms, although SV-SM showed a nominally greater association with rumination and pDMN+ with depressive symptoms [36]. In addition, among these same rMDD adolescents, we have also demonstrated reduction in residual depressive symptoms and rumination tendencies after eight weeks of a randomized pilot study of Rumination-Focused Cognitive Behavior Therapy (RFCBT) compared to an Assessment Only (AO) control to prevent depressive relapse [37].

The current report details the post-intervention rumination induction compared to Baseline, and relation to two-year clinical follow-up of these adolescents. Using changes from Baseline to post-intervention in neural activation during rumination induction (versus distraction), we explore potential brain-based treatment changes as potential predictors of relapse. This methodology allows for an exploratory investigation of whether directly targeting rumination among a population of vulnerable adolescents predicts sustained wellness over time, and whether this particular intervention facilitates neural changes that support wellness. We hypothesized that adolescents who received RFCBT would maintain lower depressive symptoms and show reduced risk to relapse relative to their AO peers over the follow-up period. Due to previously observed associations of pDMN+ activation during rumination induction with baseline depressive symptoms, we further hypothesized that activation changes in pDMN+ regions during rumination induction would predict depression symptoms prospectively. Specifically, we expected to find evidence of a disease modification model, such that decreased activation in pDMN+ regions at Week Eight would predict lower levels of depression symptoms prospectively, and a remediation effect of RFCBT. Here the hypothesis is that RFCBT *changes* brain function, to remediate ruminative, disease-associated abnormalities. As an alternative hypothesis, wellness in the current sample may be supported through compensation for disease-related brain functioning, such that RFCBT may help sustain compensation, possibly through sustained differential engagement of the pDMN+.

## Materials and methods

### Participants and procedures

Adolescents, aged 12–18, were recruited from an urban academic medical center and the surrounding community. They were required to have met full DSM-IV criteria [38] for MDD in the past, and to be currently in full or partial remission (rMDD), as reported in Jacobs et al. 2016 [37]. Partial remission was defined as no more than three threshold symptoms of MDD subsequent to a full remission, defined as the absence of any significant symptoms for at least two weeks. Full remission was defined as the absence of any clinically significant depressive symptoms (i.e., a score of three on any KSADS-PL MDD symptom). Thirty-three adolescents were enrolled and randomized to AO or RFCBT (Intent-To-Treat Sample); twenty-nine completed one fMRI scan prior to treatment randomization and another fMRI scan at Week Eight (fMRI Completer Sample). Randomization was generated using Research Randomizer stratified by sex and age. This sample was used for all clinical analyses to enhance interpretation of brain-behavior relationships. Twenty-five of these adolescents had high quality fMRI and acceptable movement parameter estimates at both timepoints, as described below, thus this sample was used for all brain-behavior analyses (Quality MRI Sample). During the two-year follow-up period, six adolescents were lost-to-follow-up or withdrew, leading to 23 adolescents in the Clinical Completer Sample. Adolescents were followed at regular intervals after the intervention period to determine the impact of RFCBT on longitudinal depressive symptoms; clinical referrals were provided for all participants who indicated a return of depressive or other clinically-significant symptoms. Measures were self-reported (by parent or adolescent; every three months) or administered by an Independent Evaluator (IE) blinded to intervention arm (every six months; see Intervention and Measures below). For the primary relapse outcome, the KSADS-PL for DSM-IV was used to establish criteria for a new episode of any mood disorder (AMD) to evaluate the broader phenotype. This category included any diagnosable unipolar depression, including dysthymia, depressive disorder not otherwise specified, and adjustment disorder with depressed mood. Relapse of a Major Depressive Episode (MDE) was also evaluated (S2 Data). The study was approved by the University of Illinois at Chicago Institutional Review Board in December 2012 (Protocol #2012–0689), and all adolescents signed assent with corresponding parent consent starting in March 2013. Recruitment occurred from March 2013 to June 2015. Trial registration occurred in July of 2013 (delay due to investigator oversight). The authors confirm that all ongoing/related trials for this intervention are registered. The current report describes results of the two-year clinical follow-up from June 2013 through June of 2017.

### Intervention and measures

RFCBT uses a functional analytic approach combined with behavioral strategies to reduce the learned habitual behavior of rumination [39]. Participants randomized to RFCBT met with a therapist (RHJ) on a weekly basis, receiving 8 sessions (45–60 minutes) of the manualized intervention adapted from the adult intervention [40], which targeted triggers, consequences, and adaptive alternative strategies to rumination. For further details on the intervention, see S2 Data or [37].

The 17-item Children’s Depression Rating Scale–Revised (CDRS-R) [41, 42] and the Schedule for Affective Disorders and Schizophrenia for School Age Children–Present and Lifetime (KSADS-PL) [43] were completed by IEs with the adolescent and a parent or legal guardian to determine current depressive symptoms and psychiatric diagnoses at Baseline, Week Eight, and every six months during follow-up. The CDRS-R is an extensively-validated measure and its depression severity score is highly reliable, externally-valid, and sensitive to change. In this study, inter-rater reliability (intraclass correlations, ICC = 98.3%) on the CDRS-R was excellent, while KSADS-PL inter-rater reliability for MDD was good (kappa>.78). Although the CDRS-R has good test-retest reliability over six week intervals [44], in the current study there was moderate two-way random test-retest reliability during the follow-up with six month intervals (ICC = 57.4%).

To assess self-reported current depressive symptoms and ruminative tendencies, adolescents completed the 30-item Reynolds Adolescent Depression Scale (RADS) [45] and the 22-item Ruminative Response Scale (RRS) [46] at Baseline, Week Eight, and every three months during the follow-up. The RADS is rated on a 4-point Likert scale and has excellent internal consistency and good retest reliability [45]; in the present study, α = .87. Higher total scores reflect higher levels of depression. One-way random test-retest reliability during follow-up was excellent (97.7%) with three-month intervals. In addition, item 14 of the RADS was used as a flag for suicidality: “I feel like hurting myself,” which can be endorsed as 1) “almost never” through 4) “most of the time.” The RRS is empirically-supported in adolescent populations [47]. In the present study, internal consistency was excellent (α = .94), as was test-retest reliability (one-way ICC) during follow-up with three-month intervals (96.0%). The brooding subscale has typically been understood as a more specific measure of rumination separate from depression severity [46], although this factor structure may not hold across depressive episodes and may correlate highly with other ruminative subfacets [48]. The brooding subscale was strongly correlated with the other subscales of the RRS across time in this sample at the level of colinearity (depression-related: *r* = .84, *p* < .001, reflection: *r* = .71, *p* < .001, total rumination: *r* = .94, *p* < .001). Adolescents also completed several other self-reports and performance measures at Baseline to characterize further the sample, including the Peterson Pubertal Development Scale [49] and the Wechsler Abbreviated Scales of Intelligence-IV, 2-Subtest [50] (Table 1).

**Table 1:** Demographics and clinical characteristics of fMRI completer sample (N = 29).

	RFCBT (*n* = 15)	AO (*n* = 14)	Statistical Test
	*M (SD) / Mdn (Range)*	*M (SD) / Mdn (Range)*	
**Age**	15.27 (2.05)	15.93 (1.77)	*t*(27) = 0.93, *p* = .36
**WASI-II Two-Test IQ**	108.86 (13.28)	109.86 (12.38)	*t*(26) = 0.21, *p* = .84
**Days Since Last MDE**	314.00 (2,852.00)	127.50 (1,110.00)	*U* = 146.00 *p* = .08
**CDRS-R Baseline**	28.00 (12.00)	25.64 (22.00)	*U* = 125.00 *p* = .40
**RADS Baseline**	63.21 (11.00)	58.08 (13.88)	*t*(27) = -1.07, *p* = .30
**RRS Baseline**	52.20 (11.57)	51.93 (13.28)	*t*(27) = 0.03, *p* = .98
	*N (%)*	*N (%)*	
**Female**	6 (40.0%)	9 (64.3%)	*ϕ* = -.24, *p* = .19
**Race/Ethnicity**			*ϕ* = .36, *p* = .43
Hispanic/Latin(x)	1 (6.7%)	4 (28.6%)	
African American/	2 (13.3%)	2 (14.3%)	
Black			
Asian	1 (6.7%)	0 (0.0%)	
Caucasian/White	8 (53.3%)	7 (50.0%)	
Other	3 (20.0%)	1 (7.1%)	
**Left Handedness**	2 (13.3%)	2 (14.3%)	*ϕ* = -.01, *p* = .94
**Additional Treatment**	11 (73.3%)	13 (92.9%)	*ϕ* = -.26, *p* = .16
**Post-Pubertal**	6 (42.9%)	9 (64.3%)	*ϕ* = .22, *p* = .26

Baseline = pre-intervention; CDRS-R = Children’s Depression Rating Scale–Revised; MDE = major depressive episode; RADS = Reynolds Adolescent Depression Scale; RRS = Ruminative Response Scale; *t* = *t*-score; *U* = Mann-Whitney; *ϕ* = phi chi-square.

### Functional imaging

#### Acquisition and preprocessing

All scans (Baseline and Week Eight) were completed on a 3.0T GE Discovery scanner (Milwaukee, WI) using parallel imaging with ASSET and T2* gradient-echo axial EPI. The following parameters were used: 90° flip, FOV = 22, MS = 64x64, ST = 3mm with 44 slices, 22.2ms ET, TR = 2,000 ms, 265 volumes for a total scan length of 200 TRs. High-resolution anatomic T1 SPGR scans were obtained for use in spatial normalization. Motion was minimized through training with a mock scanner, reminders to participants about the importance of staying still, foam pads, and a cross on the screen display.

Preprocessing included multiple steps to reduce potential sources of noise and artifact, including motion. Slice-timing was corrected with SPM8, and motion-detection algorithms using FSL’s MCFLIRT [52]. Structural images were coregistered to functional images before spatial normalization of the T1-SPGR to the Montreal Neurological Institute (MNI) template. Resulting normalization matrices were then applied to the slice-time-corrected, physiologically-adjusted time-series data. Normalized T2* time-series data were then spatially smoothed with a 5 mm Gaussian kernel, resulting in T2* images with isotropic voxels, 2 mm per side. Movement was addressed through visual examination of normality plots of average standard deviations of movement values for outliers. Individual participants with any TR to TR movement or three consecutive TRs exceeding 1.5 mm in any direction were removed from their respective models. Twenty-five adolescents had quality scan data on the rumination task at both Baseline and Week Eight. Two participants were excluded due to use of different scan parameters and two were excluded for extreme outlier movement. These four adolescents did not significantly differ on any Baseline clinical or demographic variables from the rest of the fMRI Completer sample (all *p*>.22).

#### Rumination task

The rumination task, described in [36], was designed with four blocks, each of which included mood, rumination, and distraction inductions. The task was based on principles of previous experimental psychology studies (i.e., [53]) as well as the work of [54]. Two weeks prior to each scan, adolescents generated four autobiographical negative life events corresponding to four categories (i.e., interpersonal pain, sad family event, sadness or frustration leading to hopelessness, and failure). Once in the scanner, adolescents were given the mood induction prompt to think about the negative life event and told to ‘Use your imagination to bring this fully into your mind and picture the event.’ Next, rumination prompts from Nolen-Hoeksema’s standard rumination protocols and those adapted for a younger population [55] were given (e.g., “think about what your feelings mean”). Moderate to strong reliability of activation of clusters from pDMN+ and SV-SM in the second and third rumination block were observed, *r* = .49 to .95, all *p*≤.01; See S2 Data and S2 Fig for additional details. Distraction induction included prompts to think about a specific object or event, such as a row of shampoo bottles. Self-report questions regarding sadness and self-focus immediately followed both rumination and distraction to ensure manipulation efficacy. Task length was 8.5 minutes. After participants finished the MRI, they were debriefed and given a short positive mood induction to ensure repair of mood.

#### Exploratory brain-behavior relationships

Brain-behavior analyses were run parallel to clinical analyses, with the underlying hypothesis that RFCBT is a disease-modifying treatment (rMDD with greatest rumination change would demonstrate reduced differences in pDMN+ from healthy control activation). Our alternative hypothesis, of disease compensation, implicates that the neural differences between rMDD and HC at Baseline might reflect compensation (or adaptation) to maintain wellness, and that RFCBT is a treatment to enhance or sustain compensation (stability of activation in pDMN+).

Two neural networks that previously were demonstrated to differ in activation between healthy and rMDD adolescents (in an overlapping sample) during Rumination-Distraction are used in the current study (S2 Data) [36]. These factors were determined based on exploratory factor analysis of extracted z-value activation from 14 clusters found to be significantly different between healthy and rMDD adolescents during Rumination-Distraction; wherein two factors explained approximately 79.0% of the variance. The current exploratory analyses utilized the pDMN+ (7 clusters comprising posterior default mode network and additional regions) in the second factor found to differ between groups [36] (S1 Fig and Table, referred to as disease markers). Our primary hypotheses were concerned with the pDMN+ factor, given significant correlations with both clinician-rated (*r* = .38) and self-reported (*r* = .28) Baseline depressive symptoms. For thoroughness, we present the same analyses using the first factor, SV-SM, in S1, S2 and S3 Data.

Activation during Rumination-Distraction from these regions was extracted from both Baseline and Week Eight contrast images. To calculate change scores, Baseline activation was subtracted from Week Eight activation in each region of interest for each participant to represent change across the intervention period. Next, an average value was calculated of all subtraction scores for the regions contributing to pDMN+ or SV-SM (change scores). For *n* clusters, the following equation describes the calculation for either pDMN+ or SV-SM:

$$\frac{\sum_{i=1}^{n=7}[Week\;Eight(Rumination-Distraction)]-[Baseline(Rumination-Distraction)]}{n}$$

### Statistical analyses

#### Power

As this was a pilot exploratory study, the sample size obtained was made possible with pilot and training grants to collect preliminary data for training and related grant submissions. In these situations, power is insufficient to obtain significant effects unless they are large effect sizes. Here the focus is to demonstrate feasibility and the ability to stably estimate effect sizes. Power was determined *a priori*, and as suggested, the sample size obtained could detect very large effect sizes for the primary analyses (Cohen’s *d* ≥ 1.12). See S2 Data for details of these estimates.

#### Missingness

Of the fMRI Completer Sample during the two-year follow-up phase, 75.2% of RRS and 71.4% of RADS total scores (75.8% of RADS suicidality flag) across the two-year follow-up were available. Finally, 75.8% of CDRS-R total scores and 77.0% of KSADS-PL were completed, influenced by those lost-to-follow-up. The amount of missing datapoints was well within the acceptable range for MRMs. See S2 Data for additional missingness information.

#### Sample characteristics

Treatment groups were compared on all baseline and demographic variables using the appropriate dimensional (*t*-test or Mann-Whitney) or binary nominal chi-square (phi) statistical tests. The Intent-To-Treat sample (*N* = 33) and two-year follow-up Clinical Completer Sample (*N* = 23) were compared with the fMRI Completer Sample (*N* = 29) on all demographic variables, to ensure similarity across included samples. Pearson’s correlations were conducted on ruminative and depressive symptoms across the two-year follow-up, per reviewer request.

#### Mixed-effects models

Mixed-effects regression models (MRMs) were conducted on the fMRI Completer Sample (*N* = 29) using SPSS v24.0 MIXED. MRMs allow for the dependencies inherent in repeated assessments, are robust to missing data, and can be used to estimate scores using group trajectories. MRMs were used to assess the effects of treatment, and treatment-by-time (quadratic terms, treatment-by-time-by-time, were initially included in the MRM and then removed when non-significant), on the RRS, RADS, and CDRS-R across two years of longitudinal follow-up, using Week Eight scores as the intercept. Week Eight was chosen as the intercept to clarify changes over the longitudinal follow-up, as changes between pre- and post-intervention have been documented elsewhere [37]. Time in weeks since Baseline was included as a fixed effect. In additional MRMs presented in S2 Data, age and sex were included as fixed effects as a sensitivity analysis. Due to the evaluation of three primary outcome measures (i.e., RRS, RADS, CDRS-R), a family-wise error rate for these three outcomes was used (*p* < .017, noted in results tables). To characterize further the effect of RFCBT, MRMs were also used on the suicidality flag item of the RADS across the two-year longitudinal follow-up. For completeness in response to reviewer suggestions about specificity of brooding rumination, an MRM was also conducted on the brooding subscale of the RRS. More detailed methods and results are presented in S2 and S3 Data.

#### Relapse

Uncertainty coefficient (UC) chi-square analyses were conducted to detect the strength of nominal variable differences in relapse (AMD and MDE) and hospitalization for suicidality or suicidal gesture among the fMRI Completer sample across the two-year follow-up [51]. Due to the high percentage of relapse in both arms, log rank tests were conducted to determine differences in survival distributions.

#### Rumination task and manipulation check

Reliability of activation across rumination inductions within the Rumination Task was examined by comparing activation beta weights (S2 Data). To ensure rumination induction manipulation was successful, repeated measures analyses of variance were conducted on youths’ reports of sadness and self-focus after each condition during the rumination induction task, using all youth with quality MRI and responses in each condition (Manipulation Check Sample, *N* = 21).

#### Brain-behavior analyses

For thoroughness, paired *t*-tests of average activation of disease-related networks (pDMN+ and SV-SM) were conducted of the Quality MRI Sample (*N* = 25) and by treatment group to document change over the intervention period (S3 Data). To document the relationship between each region comprising pDMN+ and SV-SM and average activation of the pDMN+ and SV-SM networks, as well as over the intervention period, pearson’s correlations were conducted.

Exploratory brain-behavior relationships were examined by testing whether change from Baseline to Week Eight in brain activation during rumination versus distraction (Rumination-Distraction) predicted Week Eight, one-year and two-year follow-up depressive symptoms with the Quality MRI Sample (*N* = 25). A series of linear regression analyses were conducted to determine whether changes in activation could predict depressive symptoms over longitudinal follow-up. Predictors included pDMN+ change scores, pDMN+ activation in Rumination-Distraction at Baseline (as a covariate to adjust for individual differences at Baseline; *r* = -.52, *p* = .01), and treatment group. The dependent variable was IE-determined depressive symptoms (CDRS-R) at three time points: Week Eight, one-year, and two-year follow-up. Because our hypotheses concerned brain-behavior relationships regardless of treatment condition, we employed effects coding, such that the intercept of the model represents the grand mean and the effect of treatment reflects change from the grand mean for the RFCBT group.

To examine exploratory brain-behavior relationships with relapse, suicidality and hospitalization, a similar strategy was used. Logistic regressions analyzed whether change scores predicted AMD and MDE relapse at one-year and two-year follow-up, and to predict suicidality and hospitalizations over the course of the study (S2 Data). Due to the high number of relapses in this sample, cox regression analyses were conducted to examine whether pDMN+ was related to survival distributions.

## Results

### Sample characteristics

**Fig 1:**
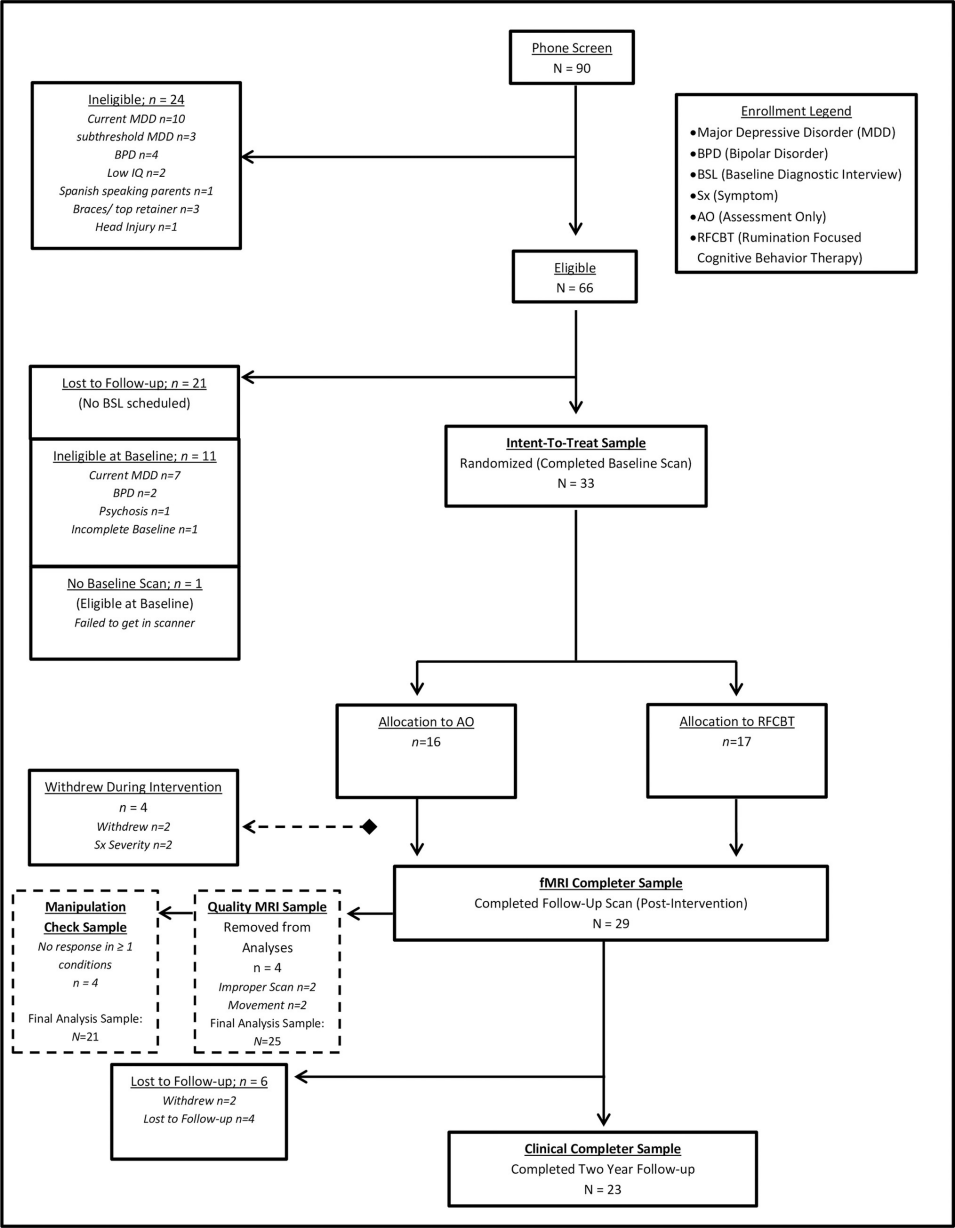
CONSORT diagram of pilot clinical trial with randomization to Rumination-Focused Cognitive Behavioral Therapy (RFCBT) or Assessment Only (AO). Brain-behavior relationship analyses were conducted among adolescents who completed follow-up scan (*N* = 29) and who were not excluded from analyses for improper scan sequences or outlier movement (*n* = 4, final *N* = 25). Clinical Completer Sample included 23 adolescents.

Table 1 presents sample demographics for the fMRI Completer Sample at Baseline. The Intent-To-Treat sample and two-year follow-up Clinical Completer Sample did not statistically differ from the fMRI Completer Sample on any demographic variables (all *p*>.42; see S2 Table). See Fig 1 for the two-year longitudinal follow-up CONSORT diagram.

Across the two-year follow-up period, self-reported depressive symptoms were weakly to moderately correlated with self-reported rumination (brooding: *r* = .52, *p* < .001; depression-related: *r* = .54, *p* < .001; reflection: *r* = .34, *p* < .001; total rumination: *r* = .52, *p* < .001).

### Mixed-effects models

#### Depressive symptoms

**Fig 2:**
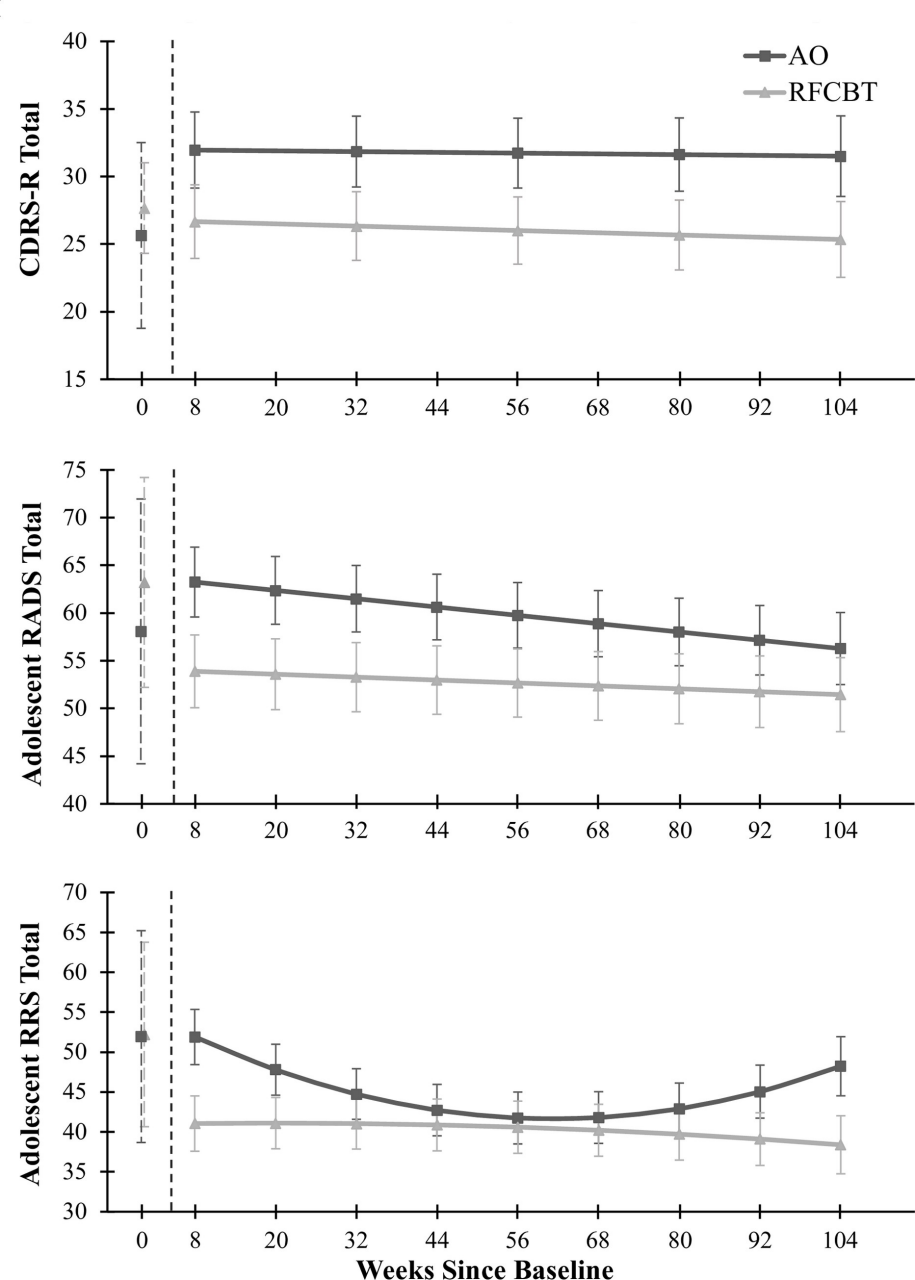
Clinical outcomes across two-years of longitudinal follow-up. Predicted means and standard errors derived from MRMs for the fMRI Completer sample. Baseline means and standard deviations are depicted for illustrative purposes and were not included in the MRM models. RFCBT = Rumination-focused Cognitive Behavior Therapy, AO = Assessment Only, RRS = Ruminative Response Scale, CDRS-R = Children’s Depression Rating Scale–Revised, RADS = Reynolds Adolescent Depression Scale. Panel a illustrates change in CDRS-R, panel b illustrates change in RADS, panel c illustrates change in RRS over two years.

This sample showed a significant intercept effect for Week Eight CDRS-R scores,reflected in the significant intercept term in the MRM model, *F*(1, 88.07) = 508.25, *p* < .001. Time, *F*(1, 99.56) = 0.25, *p* = .62, and the interaction of group-by-time, *F*(1, 99.56) = 0.06, *p* = .81, were not significant over the two-year follow-up. The AO group illustrated higher CDRS-R scores across the two-year post-intervention period when compared with the RFCBT group *F*(1, 88.07) = 4.01, *p* < .05, displayed in Fig 2A, although this did not meet the FWE-rate. See Table 2 for estimates of these fixed effects.

**Table 2:** Estimates of fixed effects for depressive symptoms (CDRS-R and RADS) over the two-year follow-up period.

Dependent Variable	B (SE)	*df*	*t*	*95% CI*
**CDRS-R**				
Intercept	26.77 (1.80)**	87.33	14.88	[23.19, 30.35]
AO Group	5.22 (2.61)	88.07	2.00	[0.04, 10.40]
Time	-0.01 (0.02)	97.94	-0.55	[-0.06, 0.04]
Time x AO Group	0.01 (0.04)	99.56	0.25	[-0.06, 0.08]
**RADS**				
Intercept	54.09 (3.35)**	43.29	16.14	[47.33, 60.85]
AO Group	9.74 (4.78)	41.97	2.04	[0.08, 19.39]
Time	-0.03 (0.03)	142.42	-0.88	[-0.08, 0.03]
Time x AO Group	-0.05 (0.04)	142.68	-1.15	[-0.13, 0.03]

B = unstandardized coefficient; SE = Standard Error; *df* = degrees of freedom; *t* = *t*-score; 95% CI = 95% Confidence Interval.

*p < .017 (FWE-rate)

**p < .005.

Similarly, the difference between the RFCBT and AO groups’ self-reported RADS depression scores across the two years post-intervention did not reach the FWE-rate for significance, *F*(1, 41.97) = 4.14, *p* < .05, displayed in Fig 2B and Table 2, although numerically in the expected direction. Over the full two-year period the AO group reported higher RADS depression scores (*M* = 59.90, *SE* = 3.09) than the RFCBT group (*M* = 52.72, *SE* = 3.01). The intercept was significant, *F*(1, 41.97) = 607.45, *p* < .001. Time (post-intervention) contributed significantly to the model, *F*(1, 142.68) = 5.67, *p* = .02, with self-reported depression scores decreasing over the longitudinal follow-up period. The interaction of group-by-time was not significant, *F*(1, 142.68) = 1.31, *p* = .25.

#### Ruminative tendencies

The RFCBT group reported lower rumination than the AO group, as measured by the RRS across the two years post-intervention, *F*(1, 63.42) = 7.21, *p* = .01. All fixed effects and interactions were significant (all *p* < .02) but qualified by the significant quadratic interaction of group-by-time-by-time, *F*(1, 167.95) = 9.01, *p* = .003, illustrated in Fig 2C and Table 3. Specifically, the AO group reported decreased rumination over time until approximately one year, as reflected in the significant group-by-time term, *F*(1, 168.78) = 9.32, *p* = .003. The quadratic interaction indicates an increase of rumination after one year as reflected in the significant group-by-time-by-time term, *F*(1, 167.95) = 9.01, *p* = .003. Although the time and time-by-time terms were significant, *F*(1, 168.78) = 8.15, *p* = .01 and *F*(1, 167.95) = 5.90, *p* = .02, respectively, the putative rebound effect seen in the AO group did not occur in the RFCBT group. See Table 3 for estimated fixed effects.

**Table 3:** Estimates of fixed effects for ruminative tendencies (RRS) over the two-year follow-up period.

Predictors	B (SE)	*df*	*t*	*95% CI*
Intercept	40.95 (3.68)**	63.27	11.12	[33.59,48.31]
AO Group	14.24 (5.30)*	63.42	-2.69	[3.64, 24.84]
Time	0.01 (0.10)	169.19	0.14	[-0.19, -0.22]
Time^2^	-0.0004 (0.001)	168.23	-0.42	[-0.002, 0. 001]
Time x AO Group	-0.46 (0.15)**	168.78	-3.05	[-0.75, -0.16]
Time^2^ x AO Group	0.004 (0.001)**	167.95	3.00	[0.001, 0.01]

B = unstandardized coefficient; SE = Standard Error; *df* = degrees of freedom; *t* = *t*-score; 95% CI = 95% Confidence Interval.

*p < .017

**p < .005.

#### Relapse

Over the two-year follow-up, relapse of AMD among the fMRI Completer sample was 86.2% (*n* = 25), whereas relapse of a major depressive episode (MDE) was 69.0% (*n* = 20). AMD relapse rates were higher in the AO group (100.0%) compared to the RFCBT group (73.3%), *UC χ*^2^(1) = 2.06, *p* = .02. Only four adolescents did not meet criteria for AMD relapse after two years.

**Fig 3:**
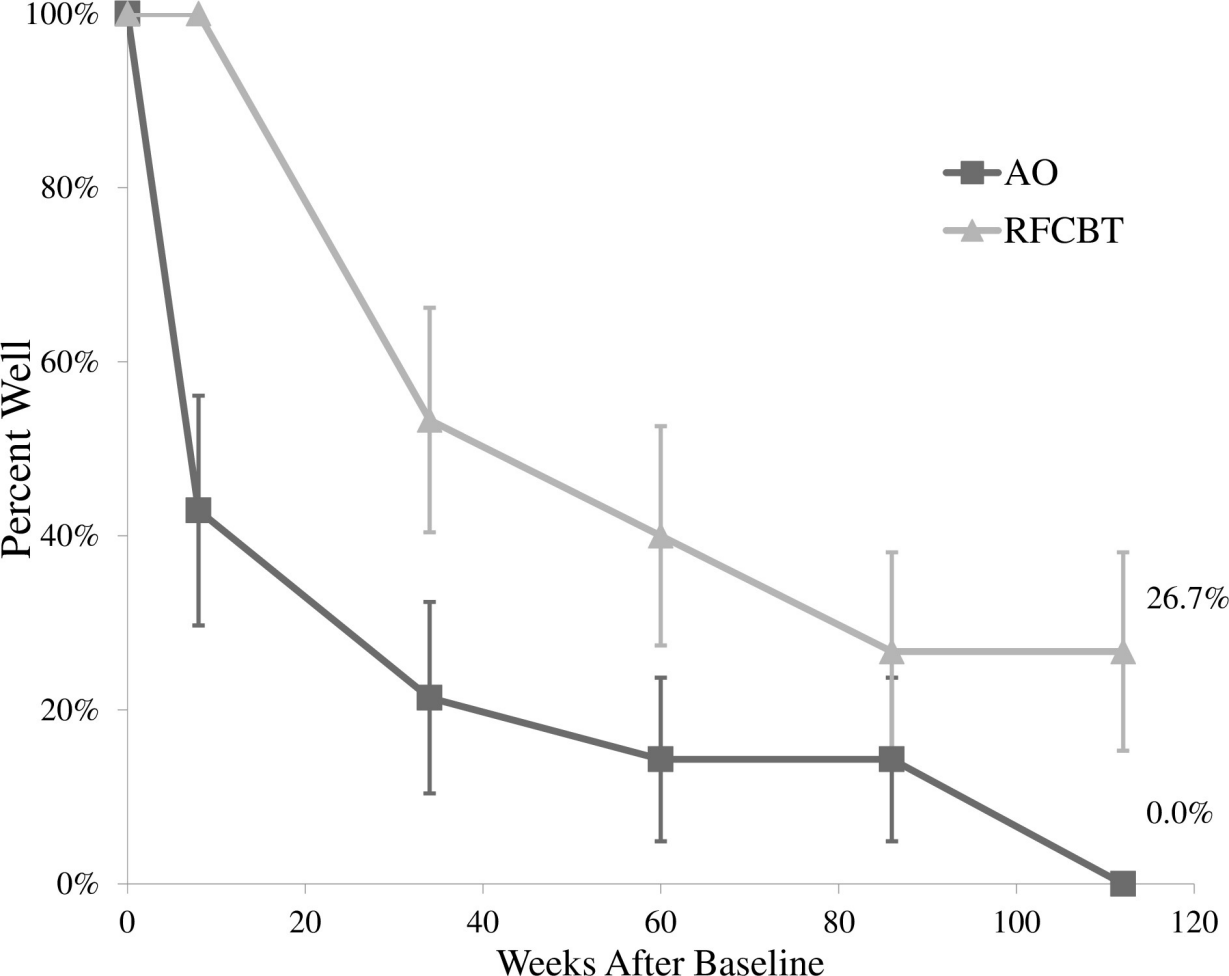
Survival plot of percent well (any mood disorder relapse) over study intervention and two-year post-intervention follow-up. RFCBT = Rumination-focused Cognitive Behavior Therapy, AO = Assessment Only, AMD = Any Mood Disorder. Percent of adolescents in each group not meeting criteria for AMD at each assessment point; at two-year study endpoint, all AO adolescents experienced a relapse, whereas 26.7% of RFCBT youth remained well.

Given such high relapse rates, a log rank test was conducted to determine differences in survival distributions. The AMD relapse distributions for the two groups differed, *χ*^2^(1) = 7.58, *p* = .01, such that on average the AO group (*M* = 32.14, *SE* = 10.00, 95% CI [12.54, 51.75]) relapsed approximately 35 weeks sooner than those in the RFCBT group (*M* = 67.43, *SE* = 8.88, 95% CI [50.03, 84.83]). See Fig 3 for a survival plot displaying the percent of adolescents who stayed well (no AMD relapse) over the course of two years, and S3 Data for suprathreshold MDE relapse rates.

#### Suicidality

Over the course of the study five adolescents in the AO group were hospitalized for suicidality or a suicidal gesture (33%), whereas no adolescents in the RFCBT group were hospitalized, *χ*^2^(1) = 7.07, *p* = .01. Over the course of the longitudinal follow-up, there was a significant difference between the AO and RFCBT groups on the suicidality flag item of the RADS as reflected in both the intercept, *F*(1, 17.97) = 4.39, *p* = .04, and group fixed effect, *F*(1, 17.97) = 4.89, *p* = .04. There were 5 separate endorsements for frequent or intense suicidal ideation in the AO versus zero in the RFCBT group. Time and the interaction of group-by-time also contributed significantly to the model, *F*(8, 139.48) = 2.41, *p* = .02; *F*(8, 139.48) = 2.41, *p* = .02 (S3 Figand S3 Data).

### Exploratory brain-behavior results

#### Rumination task and manipulation

All brain-behavior analyses were restricted to the 25 adolescents with quality MRI scans at Baseline and Week Eight. The rumination task demonstrated medium to large correlations of activation over blocks, suggesting a reliable signal (S3 Data). Youth did not report significantly different sadness, *F*(1,20) = 0.03, *p* = .87, nor self-focus, *F*(1,20) = 0.03, *p* = .87, between conditions of the Rumination Task. However, manipulations were in the direction expected: youth reported higher ratings during the rumination (sadness: *M* = 2.86, *SD* = 0.69; self-focus: *M* = 3.36, *SD* = 0.46) compared to the distraction condition (sadness: *M* = 1.83, *SD* = 0.59, Cohen’s *d* = 1.61; self-focus: *M* = 2.47, *SD* = 0.65, Cohen’s *d* = 1.60). When treatment group was included as a between-subjects factor, these results held (S2 and S3 Data).

#### Relationships of networks

In the current sample, pDMN+ and SV-SM were highly correlated across all rMDD adolescents at Baseline, *r* = .95, *p* < .001, and at Week Eight, *r* = .95, *p* < .001. As expected, each factor did not significantly correlate with itself over time, (pDMN+: *r* = .25, *p* = .22; SV-SM: *r* = -.31, *p* = .14), suggesting notable individual differences in changes in activation over the intervention period (S6 Table). This was expected as nearly all individuals were exposed to additional treatment and/or changed in symptoms and rumination over the intervention period, differing in who was exposed to RFCBT. Our hypothesis was to observe systematic change in activation for rumination induction specific to the RFCBT arm. Characteristics of each factor in each group are presented in S7 Table.

#### Depressive symptoms

**Fig 4:**
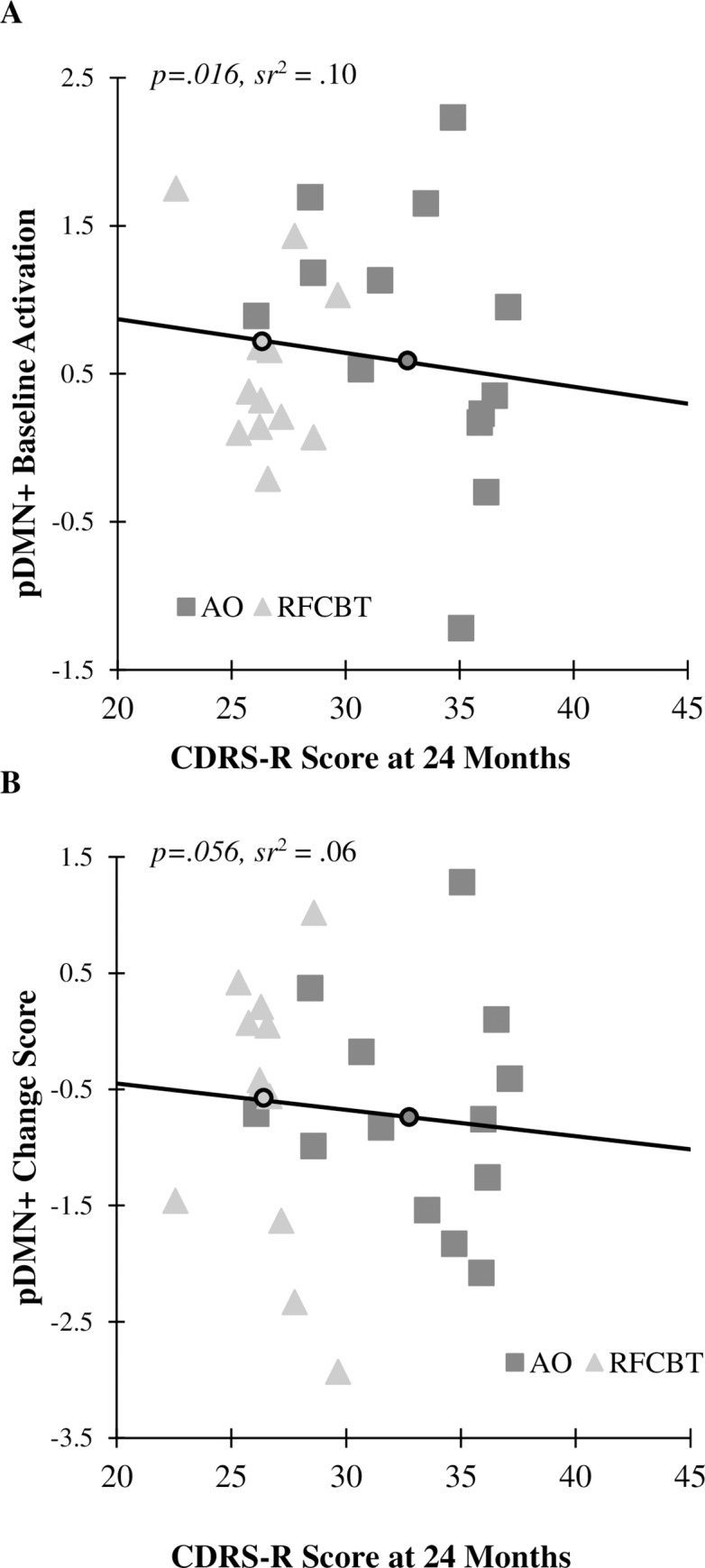
Relationship between CDRS-R depression scores at two-year follow-up with (A) Baseline activation in pDMN+ in rumination versus distraction, and (B) change in pDMN+ activation from Baseline to Week Eight during rumination versus distraction. Dark squares represent AO, whereas light triangles represent RFCBT. The solid black line represents this association across all youth. Small circles on the black line represent CDRS-R group averages at 24 months for AO (dark) and RFCBT (light). *sr*^2^ = semipartial correlation squared.

As presented in Table 4, higher Baseline activation in pDMN+ during Rumination-Distraction significantly predicted lower CDRS-R depression symptoms at Week Eight, *B* = -2.15(0.82), *p* = .02; one-year, *B* = -2.15(0.82), *p* = .02; and two-year follow-up, *B* = -2.15(0.82), *p* = .02, beyond the significant effect of treatment (RFCBT exhibited lower CDRS-R scores than AO). Fig 4 displays the change score in pDMN+ activation during Rumination-Distraction as it relates to CDRS-R score at two-year follow-up in both groups. A greater change score (more decline in activation during rumination induction versus distraction over eight weeks) was associated with higher CDRS-R depression score at two years.

**Table 4:** Activation in pDMN+ during rumination induction at baseline predicts CDRS-R depression at week eight, one-year, and two-year follow-up.

Predictor	*B* (*SE*)	*ß*	*p*		*95% CI of B*	*sr*
	*Week Eight*					
**Intercept**	29.46 (0.52)		< .001		[28.38, 30.55]	
**Treatment**	-7.74 (1.05)	-.84	< .001		[-9.93, -5.55]	-.83
**Δ pDMN+**	-1.22 (0.60)	-.27	.06		[-2.47, 0.03]	-.23
**Baseline pDMN+**	-2.15 (0.82)	-.35	.02		[-3.85, -0.45]	-.30
**Model Summary**		*F*(3, 21) = 19.06, *p* < .001; Adj. *R*^2^ = .69				
	*One-Year*					
**Intercept**	28.97 (0.52)		< .001		[27.89, 30.06]	
**Treatment**	-3.34 (1.05)	-.53	.01		[-5.53, -1.14]	-.52
**Δ pDMN+**	-1.22 (0.60)	-.39	.06		[-2.47, 0.03]	-.34
**Baseline pDMN+**	-2.15 (0.82)	-.51	.02		[-3.85, -0.45]	-.44
**Model Summary**		*F*(3, 21) = 5.17, *p* = .01; Adj. *R*^2^ = .34				
	*Two-Year*					
**Intercept**	29.83 (0.52)		< .001		[28.74, 30.91]	
**Treatment**	-6.92 (1.05)	-.81	< .001		[-9.11, -4.73]	-.80
**Δ pDMN+**	-1.22 (0.60)	-.29	.06		[-2.47, 0.03]	-.25
**Baseline pDMN+**	-2.15 (0.82)	-.38	.02		[-3.85, -0.45]	-.32
**Model Summary**		*F*(3, 21) = 15.57, *p* < .001; Adj. *R*^2^ = .65				

Brain-behavior regressions used effects coding, such that the intercept represents the whole sample, whereas treatment represents the RFCBT group compared to the assessment only group within the Quality MRI Sample (*N* = 25). B = unstandardized coefficient; SE = Standard Error; *ß* = beta; *sr* = semipartial correlation; df = degrees of freedom; 95% CI = 95% Confidence Interval; pDMN+ = posterior default mode network and additional regions.

#### Relapse

Given high AMD relapse rates (86.2%), a cox regression survival analysis was conducted to determine whether pDMN+ change scores and Baseline pDMN+ activation in Rumination-Distraction predicted time to relapse. Similar to previous analyses, treatment predicted time to relapse, χ^2^(1) = 6.42, *p* = .01, such that RFCBT was associated with lower risk of relapse (*HR* = 0.31, *p* = .02, 95% CI [0.12, 0.80]). However, pDMN+ Baseline and change scores did not predict time to relapse, Δχ^2^(2) = 1.20, *p* = .55 (*p* = .46 and .29, respectively). See S3 Data for suprathreshold MDE relapse using these brain predictors.

## Discussion

This study is a proof of concept exploration of whether therapeutic modulation of the neural networks supporting rumination protects adolescents from depressive relapse over the long-term. Youth who were randomized to RFCBT maintained lower CDRS-R scores over time and relapsed later and at a lower rate than adolescents in the AO group. Differences in self-reported depression were maintained over two years. Self-reported rumination across groups was more similar at one-year and differed in expected directions at two-year follow-up. Importantly, fewer adolescents in the RFCBT group relapsed compared to their AO peers, even at two years. Preliminary results also suggest that RFCBT may function to reduce adverse events such as hospitalizations and suicidal ideation among youth. Thus, the preliminary clinical evidence for the feasibility and utility of RFCBT in protecting adolescents over the long term is promising and worthy of future study.

The exploratory fMRI data suggest an intriguing story regarding potential mechanisms of reduced risk for depressive relapse through treatment. We hypothesized that RFCBT could function to modulate neural and emotional responses to induced active rumination, and therefore modify or change an important disease risk process (i.e., disease modification). Indeed, recent work has suggested repetitive transcranial magnetic stimulation reduces depressive symptoms through normalization of DMN hyperconnectivity [56].

Importantly, the functional imaging task used in this study *induces deliberate and voluntary* rumination in all participants, confirmed by self-reported manipulation checks. All participants are instructed to engage in this information processing style in this task, regardless of depressive symptoms or receipt of treatment targeting ruminative responses. Yet, increased activation of extensive DMN and additional regions during this rumination induction at Baseline, initially interpreted as disease markers due to increased activation compared to controls [36], in fact predicted lower depressive symptoms over the following two years. Higher Baseline activation in the SV-SM factor also predicted lower depressive symptoms over those two years. This effect was nearly doubled in adolescents who received RFCBT. Interestingly, many of the regions comprising this pDMN+ factor included DMN regions that showed greater activation during a very similar rumination induction task between depressed adults and healthy controls in a separate study [26], including PCC and PHG.

Therefore, it is possible that these results may suggest a disease compensation process which furthers this mechanism of continued remission. RFCBT may strengthen natural resilience in some and reduce depressive relapse risk in other youth by enabling management and compensation for the neural processes that underlie rumination. For instance, adolescents receiving RFCBT may have learned better strategies to stop deliberately induced rumination from filtering into the distraction period of the task, to stop expansion into uncontrolled habitual rumination, or to embrace and tolerate a brief ruminative period (such as within a scanner environment) with less distress. Indeed, there is some concurring evidence that RFCBT helps individuals with natural tendencies for depressive relapse to reduce the burden of recurrent depression [37, 40, 57]. It is possible individuals in the AO group migrated to a more avoidance-based strategy for processing aversive ruminative probes. Of course, future studies comparing RFCBT with an active, non-rumination focused strategy to prevent relapse can more directly test this hypothesis.

Early-onset depression is typically marked by more severe, comorbid, and recurrent episodes throughout the lifespan [58–60]. Thus, it is noteworthy that adolescents who received RFCBT in this study had a MDE relapse rate (53.3%) parallel to relapse rates following recovery for adolescents in the Treatment for Adolescents with Depression Study (TADS; 47%) [8]. However, adolescents in the current sample relapsed on average 64 weeks later than in the TADS longitudinal follow-up. This evidence may also support the hypothesis that RFCBT may be a disease accommodating treatment, working to enable adolescents to manage disease risk factors (e.g., habitual rumination) and diminish the impact of triggers and stressors, compared to a “curative” response wherein adolescents no longer have these disease risk factors or change to “more adaptive” brain patterns.

Of note, individual differences in activation during the rumination induction task in the SV-SM factor (salience and somatomotor networks) at Baseline also predicted decreased depressive symptoms over two years. Activation changes in SV-SM from Baseline to Week Eight did not significantly predict depressive symptoms or relapse. The regions included in the SV-SM factor also overlap with several visual processing regions that exhibited stronger activation during rumination in controls compared to depressed adults in a prior study [26]. However, in this study various prefrontal regions and the cingulate (albeit dorsal compared to subgenual anterior), also included in our SV-SM factor, showed greater activation in depressed adults than controls. Considering the strong effect of individual differences at Baseline, rumination-induced activation in these regions may represent a more trait-based mechanism of negative repetitive thought. It is also possible that change in both SV-SM and pDMN+ factors are involved in continued remission, and our sample size was too small to detect this pattern. The specificity of network activation and change during rumination induction requires empirical and direct hypothesis testing.

These analyses were considered preliminary due to limited power, given the sample size. The modeling strategy at the level of the brain was intended to illustrate how different hypotheses about the mechanisms of treatment could be tested. The results obtained, which did not support our primary hypothesis of disease modification, may be spurious or may suggest an alternative account of disease compensation. The activation patterns obtained in the Rumination Task could not be examined for test-retest reliability, given the nature of the study’s intervention targeting rumination which occurred between scan sessions. Test-retest reliability will need to be examined in another (larger) sample without an intervention. Supplemental exploratory sensitivity analyses suggested results with ruminative tendencies may be robust to sex differences, although depressive symptoms in the current sample were not. Given the robust evidence for sex and age differences in rumination and depression [61], these will be two important aspects to consider in future RFCBT studies. We look forward to larger studies that can further explore the influence of these factors.

This sample endorsed non-linear change in rumination over the longitudinal follow-up, rarely reported in other clinical trials. It is possible that this quadratic effect is an artifact of the small sample size or a result of demand characteristics. Alternatively, most adolescents in the AO group were also receiving some form of outpatient psychotherapeutic treatment, so it is possible that more standard psychotherapy treatments *indirectly* address ruminative tendencies, even if temporarily. Finally, with small samples such as this, it is always possible that treatment randomization failed and differences reflect ascertainment biases. Future studies can include the use of more varied and ecologically-valid assessments of ruminative tendencies, greater oversight of additional treatment engagement, measurement of additional important environmental factors, inclusion of transdiagnostic symptom measures, and larger samples.

In conclusion, RFCBT may capitalize upon a specific transdiagnostic method or mechanism for reduced risk for depressive relapse. Direct examination with larger samples can address this question by comparing RFCBT to another active therapeutic treatment (that does not address rumination and related negative repetitive cognitive habits, such as relaxation [40]). Importantly, prior evidence suggests that not all adolescents with a history of depression will relapse [2]. With an active treatment comparator, future studies could more readily address which adolescents are naturally resilient to relapse and thus do not require additional therapeutic interventions for relapse prevention. In contrast, if RFCBT reduces risk for recurrence over two-year follow-up via a *facilitated* resilience, then it will be important to understand the neural signature of natural *and* facilitated resilience, their differences, and how both may be enhanced or stimulated in supplementary ways (e.g., repetitive transcranial magnetic stimulation, prevention efforts). The newer NIMH R61/R33 mechanism to evaluate behavioral interventions offers such an opportunity to pursue many of these important questions.

## Supporting information

S1 Checklist CONSORT 2010 checklist of information to include when reporting a randomised trial*. (DOC)

S1 Data (DOCX)

S2 Data [62–67].
(DOCX)

S3 Data (DOCX)

S1 Fig Activation during rumination versus distraction differed in remitted depressed youth and healthy controls. Sagittal view with x slice marked. Color bar indicates *t*-test scale. Regions of significantly greater activation during Rumination-Distraction in youth with remitted major depressive disorder, relative to healthy controls.
(TIF)

S2 Fig Average activation correlation across second and third rumination block from pDMN+ and SV-SM. pDMN+ = posterior default mode and additional regions; PHG = parahippocampal gyrus; SV-SM = salience and somatomotor network factor.
(PDF)

S3 Fig Significant endorsement of suicidality flag on the RADS over the course of the longitudinal follow-up in fMRI Completer sample (*N* = 29). Predicted likelihood is plotted and spline interpolation used to clarify linearized function. Blue = AO, Green = RFCBT. Asterisks represent significant effect of time in the AO group, such that AO showed significant suicidal endorsement at Week 20 and Week 92 compared to their study endpoint. AO = assessment only; KSADS = Kiddie Schedule for Affective Disorders and Schizophrenia for School-Age Children; RFCBT = rumination-focused cognitive behavior therapy; RADS = Reynolds Adolescent Depression Scale.
(TIF)

S4 Fig Predicted estimates for brooding subscale of RRS across two years of longitudinal follow-up. Predicted means and standard errors derived from MRM for the fMRI Completer sample on the brooding subscale over two years. Baseline means and standard deviations are depicted for illustrative purposes and were not included in the MRM model. AO = assessment only; RFCBT = rumination-focused cognitive behavior therapy; RRS = Ruminative Response Scale.
(PDF)

S1 Table Foci of greater activation during rumination versus distraction in rMDD. The last column denotes which factor each region loaded onto and thus was used for the current analyses. BA = Brodmann area; HC = healthy control; k = cluster size; pDMN+ = posterior default mode and additional regions; PHG = parahippocampal gyrus; MNI = Montreal Neurological Institute space (x, y, z); rMDD = remitted major depressive disorder; Z = *z*-score peak intensity.
(DOCX)

S2 Table Demographic and clinical characteristics of Clinical Completer Sample (*N* = 23). AO = assessment only; Baseline = pre-intervention; CDRS-R = Children’s Depression Rating Scale–Revised; MDE = Major Depressive Episode; RADS = Reynolds Adolescent Depression Scale; RFCBT = rumination-focused cognitive behavioral therapy; RRS = Ruminative Response Scale; *t* = *t*-score; *U* = Mann-Whitney; *ϕ* = phi chi-square. * p < .05, two-tailed, between treatment groups in the Clinical Completer sample, such that AO reported lower depressive symptoms at Baseline.
(DOCX)

S3 Table Estimates of fixed effects when controlling for sex and age for clinician-determined, self-report depressive symptoms (CDRS-R and RADS) and ruminative tendencies (RRS) over the two-year follow-up period. AO = assessment only; CDRS-R = Children’s Depression Rating Scale–Revised; RADS = Reynolds Adolescent Depression Scale. *p < .05 **p < .005.
(DOCX)

S4 Table Estimates of fixed effects for brooding subscale of RRS over the two-year follow-up period. AO = assessment only; RRS = Ruminative Response Scale. *p < .05, **p < .005.
(DOCX)

S5 Table Characteristics by treatment group for self-reported ratings of sadness and self-focus during each condition of the rumination induction task in the Manipulation Check Sample (*N* = 21). Four youth had incomplete responses on ratings either at Baseline or Week Eight due to computer/task malfunction or slow response, leaving a total of 21 youth for these analyses (AO *n* = 10, RFCBT *n* = 11). AO = assessment only; RFCBT = rumination-focused cognitive behavioral therapy.
(DOCX)

S6 Table Correlations between all regions different from healthy controls across Baseline and Week Eight. Conducted using pearson’s correlations. pDMN+ = posterior default mode and additional regions; SV-SM = salience and somatomotor network factor from [36].* *p* < .05, two-tailed.
(DOCX)

S7 Table Characteristics of two neural factors in activation during rumination versus distraction in the Quality MRI sample and across treatment groups (*N* = 25). AO = assessment only; RFCBT = rumination-focused cognitive behavioral therapy; pDMN+ = posterior default mode and additional regions; SV-SM = salience and somatomotor network factor from [36].
(DOCX)

S8 Table Baseline activation in SV-SM during rumination induction task predicts CDRS-R depression at Week Eight, one-year, and two-year follow-up. Categorical effect of treatment using effects coding. All other predictors represent the effect of the predictor on the average of the whole sample. SV-SM = Salience and somatomotor network Factor 1 from [36].
(DOCX)

S9 Table Activation in SV-SM does not significantly predict relapse over the following two years. Hierarchical cox regressions were used to model the added benefit of SV-SM neural activation to predict relapse beyond the effect of treatment. This additional step was not significant in prediction of AMD nor MDE. AMD = any mood disorder, MDE = major depressive episode, SV-SM = salience and somatomotor network.
(DOCX)

S1 File (DOCX)
