# Supplementary material for: Malleability of rumination: An exploratory model of CBT-based plasticity and long-term reduced risk for depressive relapse among youth from a pilot randomized clinical trial
Source: PLoS One. 2020 Jun 17;15(6):e0233539. doi: 10.1371/journal.pone.0233539 (PMC7299403; doi:10.1371/journal.pone.0233539)
Supplement: S1 Data — (DOCX) [file pone.0233539.s002.docx]

**S1 Supporting information**

**Additional hypotheses**

For full transparency, all analyses using the pDMN+ factor were also conducted with the SV-SM disease-related factor as well. Although abnormal DMN connectivity has been substantially replicated in MDD across the lifespan and in relation to ruminative processes, less work has linked somatomotor and visual processing regions to MDD (although see [23] linking with rumination). However, there has been substantial evidence of involvement of aberrant salience and emotion processing networks in processes indicated in MDD, including rumination [20, 22]. As the two factors were highly correlated in the original report and nearly perfectly correlated in this subsample, we expected that SV-SM would be similar to pDMN+, but weaker and less specific.
