# Supplementary material for: Malleability of rumination: An exploratory model of CBT-based plasticity and long-term reduced risk for depressive relapse among youth from a pilot randomized clinical trial
Source: PLoS One. 2020 Jun 17;15(6):e0233539. doi: 10.1371/journal.pone.0233539 (PMC7299403; doi:10.1371/journal.pone.0233539)
Supplement: S2 Data — [62–67]. (DOCX) [file pone.0233539.s003.docx]

**S2 Supporting information**

**Supplemental methods**

**Inclusion and exclusion criteria**

Eligibility was assessed using the Kiddie Schedule for Affective Disorders and Schizophrenia for School-Age Children-Present and Lifetime Version (KSADS-PL) [43]. Adolescents were not eligible if they had a Children’s Depression Rating Scale –Revised (CDRS-R) score higher than 45 or endorsed current suicidality with plan or intent. Participants were allowed to continue all treatment including psychotherapy and medication; however, adolescents were required to be stabilized on medication, which was defined as taking an antidepressant (selective serotonin/serotonin-norepinephrine reuptake inhibitors) for a minimum of 12 weeks with no dose changes within the two weeks prior to enrollment. In addition to subthreshold or threshold MDD, exclusion criteria included: a Wechsler Abbreviated Scale of Intelligence 2-Factor Score (WASI IQ)<70 [50], a primary diagnosis of another Axis I or II DSM-IV disorder other than ADHD, or psychotropic medication other than a stimulant for ADHD or selective serotonin/serotonin-norepinephrine reuptake inhibitor antidepressant (i.e., mood stabilizers). Comorbidity was not exclusionary if secondary to MDD with the exception of a lifetime history of autism, psychotic disorder, mania, eating disorder, or alcohol/substance abuse or dependence within the previous six months. Additional exclusions to ensure MRI safety included: presence of metal in the body (such as braces), claustrophobia, and current pregnancy.

**Intervention**

RFCBT adopts a functional analytic approach to the habitual learned behavior of rumination [14]. Adolescents randomized to RFCBT [40] were taught to notice triggers and consequences of rumination, as well as learning how to shift into practicing more adaptive strategies, such as behavioral activation, active problem-solving, and mindfulness.

**Randomization and drop-out**

The CONSORT diagram for the pilot randomized controlled trial has been previously published, and is reprinted in Fig 1 [37]. Thirty-three participants (Intent-To-Treat Sample) were randomized to RFCBT (*n*=17) or Assessment Only (AO; *n*=16), stratified by age and sex. Participants were informed of their randomization after completing the Baseline scan. Four adolescents (2 RFCBT; 2 AO) of the original 33 were withdrawn during the intervention period due to increasing symptoms and need for intervention or running away from home, and thus did not receive a Week Eight MRI scan (Fig 1). Twenty-nine adolescents completed the follow-up scan (fMRI Completer Sample); of these, four were excluded from brain-behavior relationship analyses due to improper scan sequences or outlier movement (*n*=4). The Clinical Completer Sample included 23 of these adolescents.

**Power**

As this was a pilot exploratory study, power analyses were calculated by using standard repeated-measures analysis of variance (RMANOVA) using G*Power 3.1 [62]. Mixed-effects models will standardly have greater power due to the ability to measure and better control for within-person variability. Test-retest reliability correlations were determined based upon previously published literature [63], with a weighted correlation determined for RRS (with *N*=1597; [64]) and CDRS-R (with *N*=236; [65-67]). Given a false discovery rate (FDR) correction to adjust for three primary outcome measures (α=.017), five measurements of the CDRS-R over the follow-up period, weighted *r*=.61, and power=.80, the pilot study was able to detect very large effect sizes (*f*>0.57) for the CDRS-R with treatment. With α=.017, nine measurements of the RADS over the follow-up period, *r*=.85, and power=.80, the pilot study was able to detect very large effect sizes (*f*>0.64) for the RADS with treatment. Finally, with α=.017, nine measurements of the RRS over the follow-up period, *r*=.85, and power=.80, the pilot study was able to detect very large effect sizes (*f*>0.56) for the RRS with treatment.

**Missingness**

Time was significantly related to missingness on the RRS, *t*(87.07)=-4.35, *p*<.001; RADS, *t*(100.86)=-4.05, *p*<.001; CDRS-R, *t*(90.15)=-4.39, *p*<.001; and KSADS completion, *t*(80.26)=-4.12, *p*<.001, such that missingness was more likely later in the follow-up period. Age was significantly related to missingness on the RADS, *t*(59.33)=-2.26, *p*=.03; CDRS-R, *t*(43.61)=-2.64, *p*=.01; and KSADS-PL completion, *t*(40.92)=-2.77, *p*=.01, such that missingness was more likely in older participants. Otherwise, Little’s MCAR suggested that data was missing at random, *χ*^2^(31)=42.76, *p*=.08.

**Neural network factors**

Two neural network factors were found to differ at Baseline testing between healthy control and remitted MDD adolescents using whole-brain *t*-tests conducted in SPM8 with the contrast of Rumination-Distraction [36]. Whole-brain correction was achieved at *p*<.05 by using 3dClustSim-corrected joint threshold of height and extent with 10,000 Monte Carlo simulations (*p*<.005, k>57). All brain-behavior analyses conducted in the current study were based upon activation in these two factors (at Baseline) from the Rumination-Distraction contrast as disease-related networks.

**Rumination Task**

This task has been previously described in detail in [36]. Adolescents were guided through four blocks of mood induction instructions, rumination prompts, self-rating questions, distraction prompts, and the same self-rating questions. Mood induction lasted for 25 s, rumination prompt for 30 s, self-rating questions for 12 s, then 10 to 15 s jittered crosshair, 30 s for distraction prompting, and finally 12 s for questions. All four blocks followed the same sequence; all adolescents obtained this same sequence. Self-rating questions were “How sad do you feel right now?” and “How much are you focused on your feelings right now?” which were answered based on a 1 (not a tall) to 4 (a lot) Likert scale. Each question was displayed for 6 s, for a total of 12 s after both the rumination and distraction trials. Adolescents obtained a positive mood induction after leaving the scanner and were queried regarding their current mood; if positive mood remained low, a clinical psychologist on call was notified to check in with the participant.

The Quality MRI Sample (*N*=25) was used to determine reliability of activation across rumination blocks. This was examined by determining the beta weight of each rumination block after removing known variance due to the other conditions; variance not described by the model was included in these beta estimates. The average of these beta estimates for each of the 7 clusters in the pDMN+ and the 8 clusters in the SV-SM were then extracted. Beta estimates for the second and third rumination blocks were used to examine inter-block correlations.
