# Supplementary material for: Malleability of rumination: An exploratory model of CBT-based plasticity and long-term reduced risk for depressive relapse among youth from a pilot randomized clinical trial
Source: PLoS One. 2020 Jun 17;15(6):e0233539. doi: 10.1371/journal.pone.0233539 (PMC7299403; doi:10.1371/journal.pone.0233539)
Supplement: S3 Data — (DOCX) [file pone.0233539.s004.docx]

**S3 Supporting Information**

**Supplemental results**

**Clinical results – covarying for age and sex**

When MRMs included predictors for mean-centered age and sex, results were largely consistent with those results reported in the manuscript, albeit occasionally reduced to nonsignificant findings potentially due to reduced power. Specifically, clinician-determined depressive symptoms (CDRS-R) reflected a significant intercept effect at Week Eight, *F*(1, 88.92)=511.76, *p*<.001, and no other significant fixed effects (all *p*>.05). Similarly, self-reported depressive symptoms (RADS) showed a significant intercept effect at Week Eight, *F*(1, 41.88)=606.93, *p*<.001, and a significant linear decreasing effect with time post-intervention, *F*(1, 142.53)=5.70, *p*=.02. No other fixed effects were significant (all *p*>.05). Finally, ruminative tendencies (RRS) showed a significant intercept effect at Week Eight, *F*(1, 69.94)=360.50, *p*<.001, a significant effect of sex such that males reported less rumination, *F*(1, 28.41)=4.22, *p*=.049, a significant group effect with higher rumination in AO, *F*(1, 65.53)=5.86, *p*=.02, and significant linear and quadratic decreasing time effects, *F*(1, 169.06)=8.17, *p*=.005 and *F*(1, 168.22)=5.86, *p*=.02, respectively. Age was not significant, *F*(1, 29.06)=0.33, *p*=.57. These rumination results were qualified by significant interactions of group with both linear and quadratic time, *F*(1, 168.97)=9.12, *p*=.003 and *F*(1, 168.16)=8.82, *p*=.003, respectively. Similar to results presented in the manuscript, AO showed a subsequent increase in rumination after one year; the RFCBT group did not show this effect. See S3 Table for estimated fixed effects.

**Clinical results – Brooding**

Results of the MRM conducted on the brooding subscale of the RRS across the two-year follow-up reflected a significant intercept effect at Week Eight, *F*(1, 69.02)=229.73, *p*<.001, and a significant linear decrease in brooding tendencies over the post-intervention time, *F*(1, 168.56)=5.03, *p*=.03. No other fixed effects met statistical thresholds for significance. Estimated fixed effects are presented in S4 Table and S4 Fig.

**Clinical results – MDE relapse**

Over the two-year follow-up, there was no difference between treatment groups when specifically examining suprathreshold major depressive episode (MDE) relapses, *UC χ*^2^(1)=1.02, *p*=.05. A total of nine adolescents did not have an MDE relapse in the longitudinal follow-up. Of those who evidenced suprathreshold MDE relapse (69.0%), time to relapse was significantly different across the two groups, *χ*^2^(1)=4.75, *p*=.03, such that AO (*M*=53.50, *SE*=13.37, 95% CI [27.30, 79.70]) relapsed on average 33 weeks sooner than RFCBT (*M*=86.00, *SE*=8.49, 95% CI [69.37, 102.63]).

**Rumination Task – Activation Reliability**

Baseline correlations between second and third blocks ranged between *r*=.62, *p*=.001, and *r*=.88, *p*<.001, with lowest from lingual occipital (SV-SM) and highest from fusiform (pDMN+) clusters. Week Eight correlations between second and third blocks was more variable, with range between *r*=.49, *p*=.01, and *r*=.95, *p*<.001, with precuneus showing the lowest and thalamus/putamen/amygdala/PHG showing the highest correlation across blocks, both from the pDMN+ factor. See S2 Fig for a graphical depiction.

**Brain-behavior results – Further manipulation checks**

Repeated measures ANOVAs were conducted on youth’s report of sadness and self-focus after rumination and distraction blocks of the rumination induction task. In addition to the effects of condition reported in the manuscript, time showed a significant effect for both sadness, *F*(1, 20) =76.01, *p*<.001, and self-focus, *F*(1, 20)=65.39, *p*<.001, such that ratings were higher at Baseline than Week Eight. The interaction of time with condition was not significant for sadness, *F*(1, 20) =0.28, *p*=.60, nor self-focus, *F*(1, 20)=1.06, *p*=.32.

When treatment group was included as a between-subjects effect in both analyses, the previous reported effects remained similar. In addition, there was a significant effect of treatment group for self-focus, *F*(1, 19)=5.08, *p*=.04, but not sadness, *F*(1, 19)=0.01, *p*=.94, such that AO reported greater self-focus than RFCBT across conditions. Treatment and time showed a significant interaction (sadness: *F*(1, 19)=4.77, *p*=.04; self-focus: *F*(1, 19)=5.08, *p*=.04), such that RFCBT showed a greater reduction in both sadness and self-focus ratings over time compared to AO. The interaction of treatment group and condition was not significant (sadness: *F*(1, 19)=1.60, *p*=.22; self-focus: *F*(1, 19)=2.06, *p*=.17), nor was the three-way interaction with time (sadness: *F*(1, 19)=0.01, *p*=.93; self-focus: *F*(1, 19)=0.06, *p*=.81). See S5 Table for rating characteristics by treatment group, condition, and time.

**Brain-behavior results – Relapse**

Cox regression analysis was conducted to determine whether pDMN+ Baseline and change scores in Rumination-Distraction, above and beyond treatment group, predicted MDE time to relapse. Addition of these covariates did not increase model fit, ∆*χ*^2^(2)=4.30, *p*=.12. Cox regression analysis using change and Baseline in SV-SM also showed no significant prediction of either AMD nor MDE relapse (all *p*>.11).

**Brain-behavior results – Suicidality**

Regression analyses were conducted to predict endorsement of the suicidality flag item on the RADS at Week Eight, one-year, and two-year follow-up based on activation in Rumination-Distraction in neural factors at Baseline and change scores. There was no significant effect of either pDMN+ or SV-SM (all *p*>.32). Similarly, logistic regressions to predict hospitalization did not show significant effects for either neural factor (all *p*>.52).

References for Supporting Information
