# Supplementary figures and images for: Malleability of rumination: An exploratory model of CBT-based plasticity and long-term reduced risk for depressive relapse among youth from a pilot randomized clinical trial
Source: PLoS One. 2020 Jun 17;15(6):e0233539. doi: 10.1371/journal.pone.0233539 (PMC7299403; doi:10.1371/journal.pone.0233539)

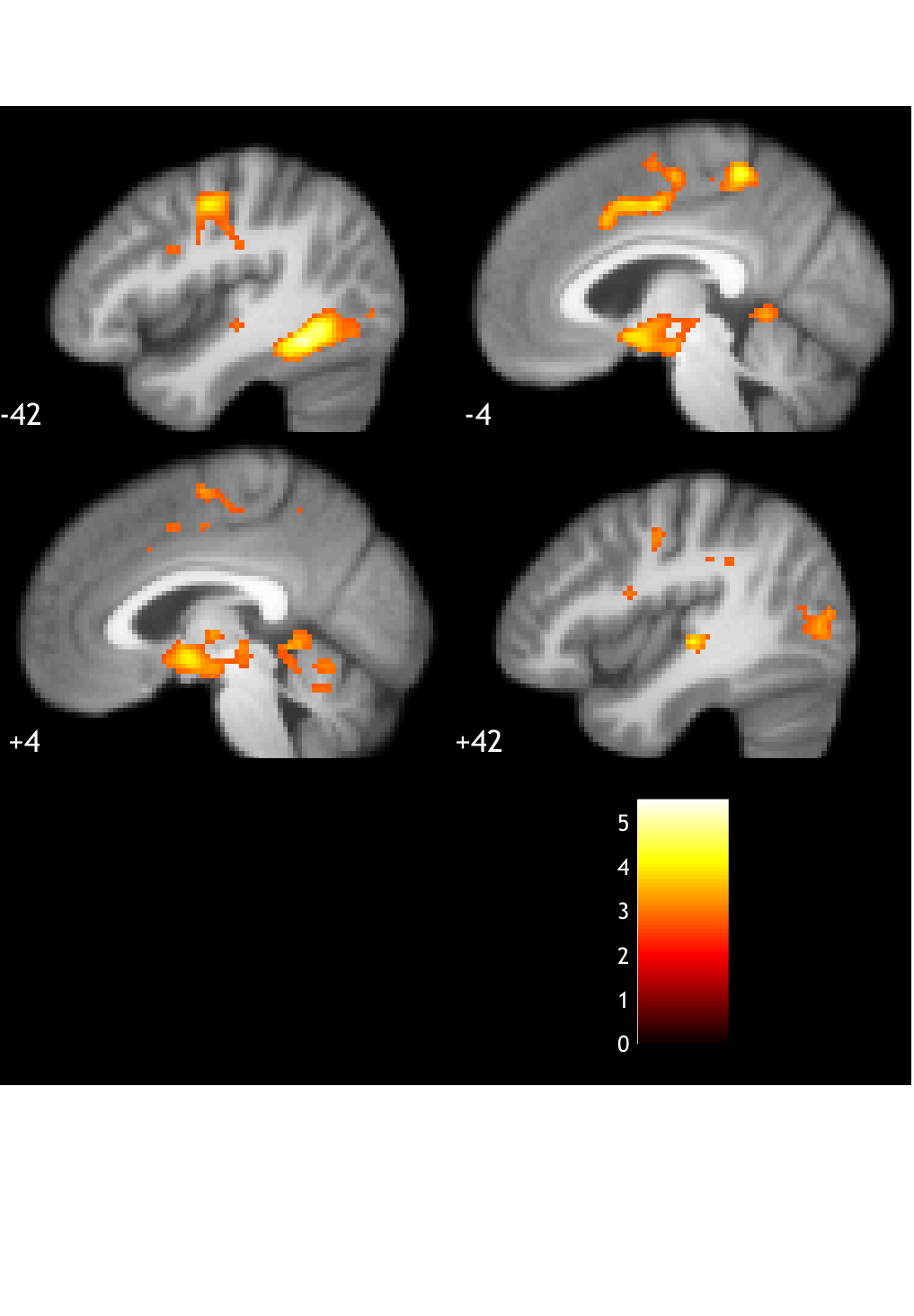

Supplement: S1 Fig — Sagittal view with x slice marked. Color bar indicates t-test scale. Regions of significantly greater activation during Rumination-Distraction in youth with remitted major depressive disorder, relative to healthy controls. (TIF) [file pone.0233539.s005.tif]

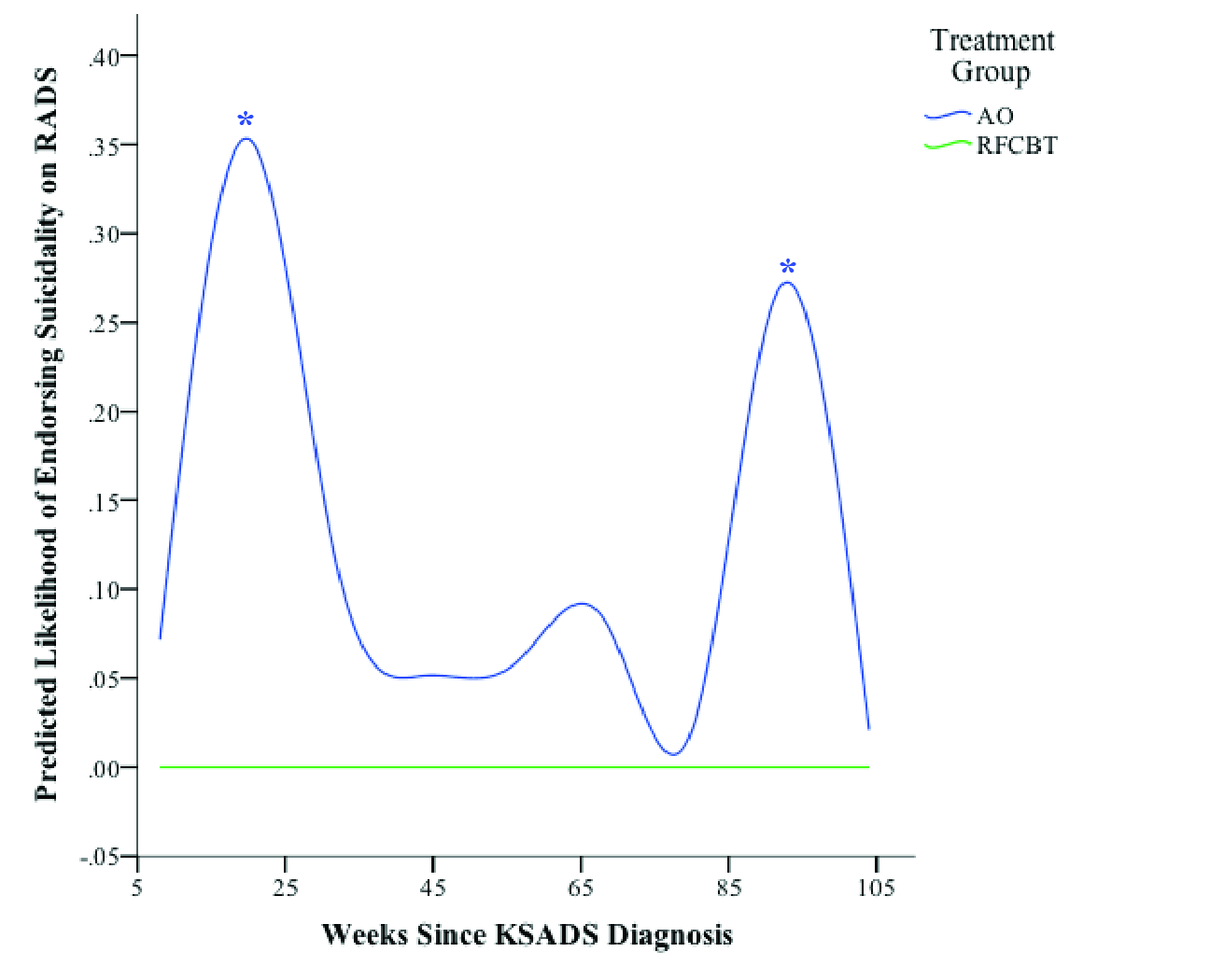

Supplement: S3 Fig — Predicted likelihood is plotted and spline interpolation used to clarify linearized function. Blue = AO, Green = RFCBT. Asterisks represent significant effect of time in the AO group, such that AO showed significant suicidal endorsement at Week 20 and Week 92 compared to their study endpoint. AO = assessment only; KSADS = Kiddie Schedule for Affective Disorders and Schizophrenia for School-Age Children; RFCBT = rumination-focused cognitive behavior therapy; RADS = Reynolds Adolescent Depression Scale. (TIF) [file pone.0233539.s007.tif]
