# Supplementary material for: Malleability of rumination: An exploratory model of CBT-based plasticity and long-term reduced risk for depressive relapse among youth from a pilot randomized clinical trial
Source: PLoS One. 2020 Jun 17;15(6):e0233539. doi: 10.1371/journal.pone.0233539 (PMC7299403; doi:10.1371/journal.pone.0233539)
Supplement: S2 Fig — pDMN+ = posterior default mode and additional regions; PHG = parahippocampal gyrus; SV-SM = salience and somatomotor network factor. (PDF) [file pone.0233539.s006.pdf]

S2 Fig. Average activation correlation across second and third rumination blocks from pDMN+ and SV-SM.

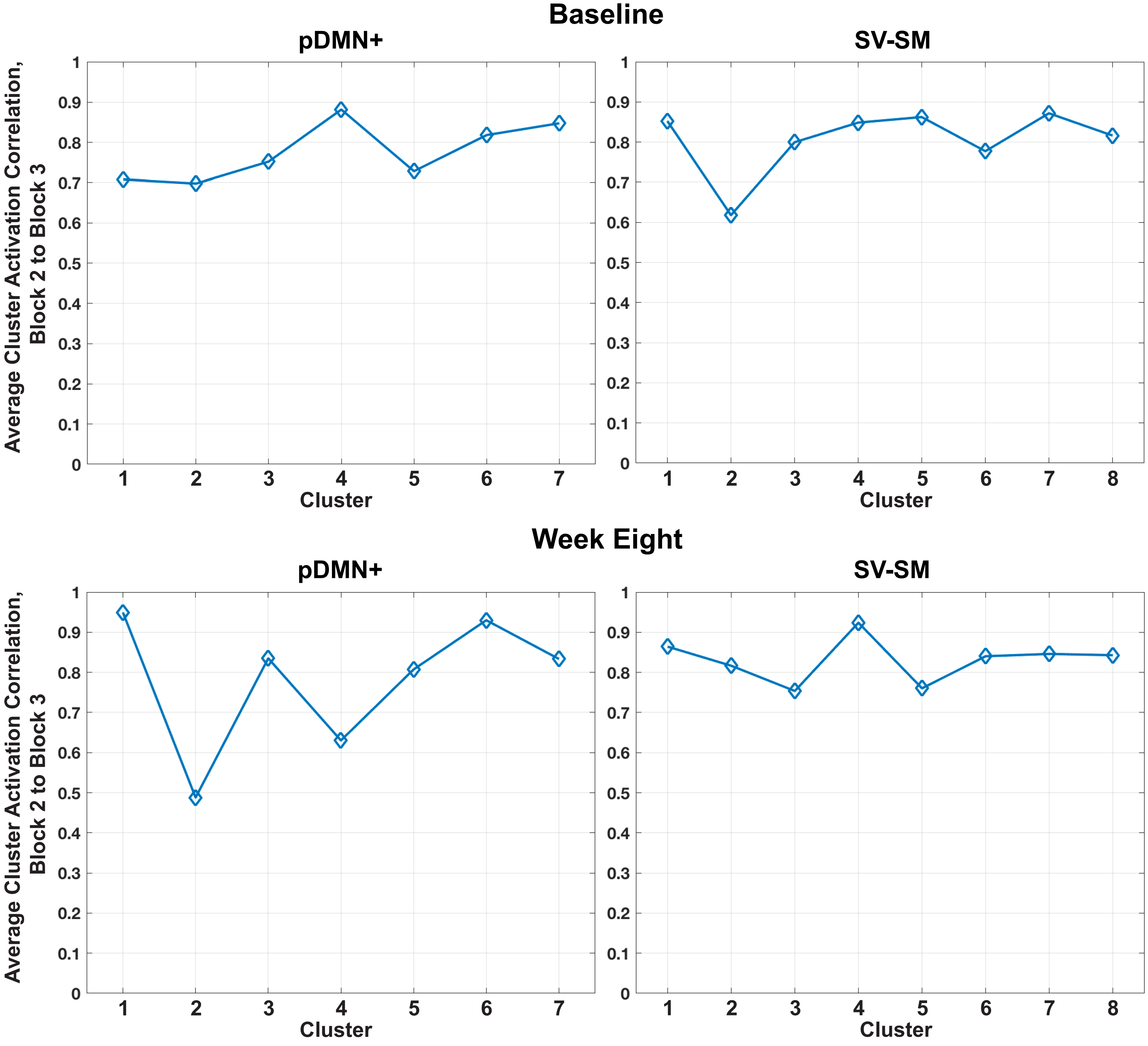

Cluster #	pDMN+	SV-SM
1	Thalamus/putamen/amygdala/PHG	Cingulate, Frontal
2	Precuneus, Parietal	Lingual, Occipital
3	Lingual, Occipital	Middle, Temporal
4	Fusiform, Temporal	Precentral, Frontal
5	Precuneus, Parietal	Insula, Subcortical
6	Superior, Temporal	Inferior, Occipital
7	Inferior, Parietal	Precentral, Frontal
8		Precentral, Frontal
