# Supplementary material for: Malleability of rumination: An exploratory model of CBT-based plasticity and long-term reduced risk for depressive relapse among youth from a pilot randomized clinical trial
Source: PLoS One. 2020 Jun 17;15(6):e0233539. doi: 10.1371/journal.pone.0233539 (PMC7299403; doi:10.1371/journal.pone.0233539)

S4 Fig. Predicted estimates for brooding subscale of RRS across two years of longitudinal follow-up.

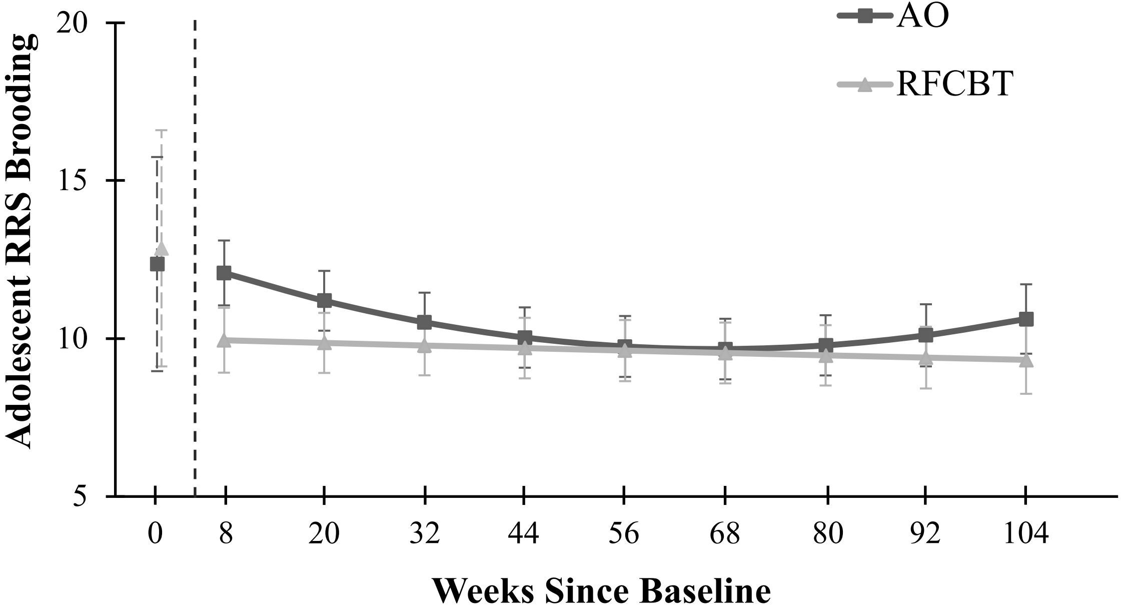

Supplement: S4 Fig — Predicted means and standard errors derived from MRM for the fMRI Completer sample on the brooding subscale over two years. Baseline means and standard deviations are depicted for illustrative purposes and were not included in the MRM model. AO = assessment only; RFCBT = rumination-focused cognitive behavior therapy; RRS = Ruminative Response Scale. (PDF) [file pone.0233539.s008.pdf]
