# Supplementary material for: Malleability of rumination: An exploratory model of CBT-based plasticity and long-term reduced risk for depressive relapse among youth from a pilot randomized clinical trial
Source: PLoS One. 2020 Jun 17;15(6):e0233539. doi: 10.1371/journal.pone.0233539 (PMC7299403; doi:10.1371/journal.pone.0233539)
Supplement: S1 Table — The last column denotes which factor each region loaded onto and thus was used for the current analyses. BA = Brodmann area; HC = healthy control; k = cluster size; pDMN+ = posterior default mode and additional regions; PHG = parahippocampal gyrus; MNI = Montreal Neurological Institute space (x, y, z); rMDD = remitted major depressive disorder; Z = z-score peak intensity. (DOCX) [file pone.0233539.s009.docx]

**S1 Table. Foci of greater activation during rumination versus distraction in rMDD.**

|  |  |  | **MNI coordinates** | | |  |  |  |  |
| --- | --- | --- | --- | --- | --- | --- | --- | --- | --- |
| **Contrast/lobe** | **BA** |  | **x** | **y** | **z** |  | **Z** | **k** | **Factor** |
| **rMDD > HC** |  |  |  |  |  |  |  |  |  |
| Frontal |  |  |  |  |  |  |  |  |  |
| Precentral | 44 |  | 56, | 6, | 10 |  | 3.88 | 544 | SV-SM |
| Precentral | 4 |  | -40, | -10, | 44 |  | 3.73 | 1007 | SV-SM |
| Cingulate | 24 |  | -2, | 4, | 44 |  | 3.85 | 1079 | SV-SM |
| Temporal |  |  |  |  |  |  |  |  |  |
| Fusiform | 19 |  | -40, | -50, | -14 |  | 4.73 | 996 | pDMN+ |
| Middle Temporal | 30 |  | -32, | -72, | 14 |  | 3.46 | 76 | SV-SM |
| Superior | 22 |  | 64, | -14, | 6 |  | 3.36 | 82 | pDMN+ |
| Parietal |  |  |  |  |  |  |  |  |  |
| Inferior | 40 |  | 66, | -34, | 34 |  | 3.71 | 474 | pDMN+ |
| Precuneus | 7 |  | -4, | -42, | 56 |  | 3.99 | 1279 | pDMN+ |
| Precuneus | 31 |  | 24, | -68, | 30 |  | 3.49 | 715 | pDMN+ |
| Subcortical |  |  |  |  |  |  |  |  |  |
| Insula | 13 |  | 40, | -22, | -2 |  | 3.82 | 279 | SV-SM |
| Thalamus/Putamen/  Amygdala/PHG | - |  | 12, | -12, | 0 |  | 3.91 | 2093 | pDMN+ |
| Occipital |  |  |  |  |  |  |  |  |  |
| Inferior | 19 |  | 46, | -72, | 4 |  | 3.88 | 212 | SV-SM |
| Lingual | 19 |  | 32, | -58, | 4 |  | 4.00 | 600 | SV-SM |
| Lingual | 19 |  | -26, | -70, | 2 |  | 3.25 | 69 | pDMN+ |
