# Supplementary material for: Malleability of rumination: An exploratory model of CBT-based plasticity and long-term reduced risk for depressive relapse among youth from a pilot randomized clinical trial
Source: PLoS One. 2020 Jun 17;15(6):e0233539. doi: 10.1371/journal.pone.0233539 (PMC7299403; doi:10.1371/journal.pone.0233539)
Supplement: S2 Table — AO = assessment only; Baseline = pre-intervention; CDRS-R = Children’s Depression Rating Scale–Revised; MDE = Major Depressive Episode; RADS = Reynolds Adolescent Depression Scale; RFCBT = rumination-focused cognitive behavioral therapy; RRS = Ruminative Response Scale; t = t-score; U = Mann-Whitney; ϕ = phi chi-square. * p < .05, two-tailed, between treatment groups in the Clinical Completer sample, such that AO reported lower depressive symptoms at Baseline. (DOCX) [file pone.0233539.s010.docx]

**S2 Table. Demographic and Clinical Characteristics of Clinical Completer Sample (*N*=23).**

|  | RFCBT  (*n*=12) | | AO  (*n*=11) | | Statistical Test |
| --- | --- | --- | --- | --- | --- |
| Characteristics | *M (SD) / Mdn (Range)* | | *M (SD) / Mdn (Range)* | |  |
| Age | 14.83 | (2.04) | 15.91 | (1.97) | *t*(21)=1.28, *p*=.21 |
| WASI-II Two-Test IQ | 109.75 | (14.21) | 109.00 | (12.09) | *t*(21)=0.36, *p*=.89 |
| Days Since Last MDE | 288.00 | (2,852.00) | 154.00 | (1,109.00) | *U*=81.00 *p*=.38 |
| CDRS-R Baseline | 27.50 | (11.00) | 22.00 | (22.00) | *U*=95.00 *p*=.08 |
| RADS Baseline* | 63.55 | (8.97) | 54.45 | (10.42) | *t*(20)=-2.19, *p*=.04 |
| RRS Baseline | 49.83 | (11.75) | 48.73 | (13.05) | *t*(21)=-0.21, *p*=.83 |
|  | *N (%)* | | *N* *(%)* | |  |
| Female | 5 | (41.7%) | 6 | (54.5%) | *φ*=-.13, *p*=.54 |
| Race/Ethnicity  Hispanic/Latin(x)  African American/  Black  Asian  Caucasian/White  Other | 0  2  1  7  2 | (0.0%)  (16.7%)  (8.3%)  (58.3%)  (16.7%) | 3  2  0  5  1 | (27.3%)  (18.2%)  (0.0%)  (45.5%)  (9.1%) | *φ*=.45, *p*=.33 |
| Left Handedness | 2 | (16.7%) | 2 | (18.2%) | *φ*=-.02, *p*=.92 |
| Additional Treatment | 8 | (66.7%) | 10 | (90.9%) | *φ*=-.29, *p*=.16 |
| Post-Pubertal | 6 | (54.5%) | 5 | (45.5%) | *φ*=.09, *p*=.67 |
