# Supplementary material for: Malleability of rumination: An exploratory model of CBT-based plasticity and long-term reduced risk for depressive relapse among youth from a pilot randomized clinical trial
Source: PLoS One. 2020 Jun 17;15(6):e0233539. doi: 10.1371/journal.pone.0233539 (PMC7299403; doi:10.1371/journal.pone.0233539)
Supplement: S3 Table — AO = assessment only; CDRS-R = Children’s Depression Rating Scale–Revised; RADS = Reynolds Adolescent Depression Scale. *p < .05 **p < .005. (DOCX) [file pone.0233539.s011.docx]

**S3 Table. Estimates of fixed effects when controlling for sex and age for clinician-determined, self-report depressive symptoms (CDRS-R and RADS) and ruminative tendencies (RRS) over the two-year follow-up period.**

| **Dependent Variable** | ***B* (SE)** | ***df*** | ***t*** | ***95% CI*** | |
| --- | --- | --- | --- | --- | --- |
| **CDRS-R** | | | | |  |
| Intercept | 27.27 (2.13)** | 60.20 | 12.80 | [23.01, 31.53] | |
| Age | -0.29 (0.49) | 25.40 | -0.60 | [-1.29, 0.71] | |
| Sex (Male) | -0.97 (1.88) | 25.75 | -0.52 | [-4.84, 2.89] | |
| AO Group | 5.20 (2.66) | 83.54 | 1.96 | [-0.09, 10.50] | |
| Time | -0.01 (0.02) | 98.06 | -0.59 | [-0.06, 0.03] | |
| Time x AO Group | 0.01 (0.04) | 99.92 | 0.26 | [-0.06, 0.08] | |
| **RADS** | | | | | |
| Intercept | 54.29 (4.35)** | 35.65 | 12.49 | [45.47, 63.10] | |
| Age | -0.27 (1.16) | 28.13 | -0.23 | [-2.65, 2.11] | |
| Sex (Male) | 0.48 (4.48) | 27.34 | -0.11 | [-9.66, 8.71] | |
| AO Group | 9.81 (4.99) | 40.31 | 1.97 | [-0.27, 19.89] | |
| Time | -0.03 (0.03) | 142.10 | -0.89 | [-0.08, 0.03] | |
| Time x AO Group | -0.05 (0.04) | 142.51 | -1.14 | [-0.13, 0.03] | |
| **RRS** | | | | | |
| Intercept | 45.82 (4.32)** | 48.90 | 10.62 | [37.14, 54.49] | |
| Age | -0.61 (1.07) | 29.06 | -0.57 | [-2.80, 1.58] | |
| Sex (Male) | -8.50 (4.13)* | 28.41 | -2.06 | [16.96, -0.03] | |
| AO Group | 12.58 (5.20)* | 65.53 | 2.42 | [2.20, 22.96] | |
| Time | 0.01 (0.10) | 169.36 | 0.12 | [-0.19, 0.22] | |
| Time^2^ | -0.0004 (0.001) | 168.37 | -0.40 | [-0.002, 0. 001] | |
| Time x AO Group | -0.45 (0.15)** | 168.97 | -3.02 | [-0.75, -0.16] | |
| Time^2^ x AO Group | 0.004 (0.001)** | 168.16 | 2.97 | [0.001, 0.01] | |
