# Supplementary material for: Malleability of rumination: An exploratory model of CBT-based plasticity and long-term reduced risk for depressive relapse among youth from a pilot randomized clinical trial
Source: PLoS One. 2020 Jun 17;15(6):e0233539. doi: 10.1371/journal.pone.0233539 (PMC7299403; doi:10.1371/journal.pone.0233539)
Supplement: S4 Table — AO = assessment only; RRS = Ruminative Response Scale. *p < .05, **p < .005. (DOCX) [file pone.0233539.s012.docx]

**S4 Table. Estimates of fixed effects for brooding subscale of RRS over the two-year follow-up period.**

| **Predictors** | ***B* (SE)** | ***df*** | ***t*** | ***95% CI*** |
| --- | --- | --- | --- | --- |
| Intercept | 10.01 (1.04)** | 68.86 | 9.59 | [7.92, 12.09] |
| AO Group | 2.77 (1.50) | 69.02 | 1.84 | [-0.23, 5.77] |
| Time | -0.01 (0.03) | 168.96 | -0.24 | [-0.07, 0.05] |
| Time^2^ | 0.00001 (0.0003) | 167.89 | 0.03 | [-0.001, 0. 001] |
| Time x AO Group | -0.09 (0.04) | 168.56 | -1.92 | [-0.17, 0.003] |
| Time^2^ x AO Group | 0.001 (0.0004) | 167.61 | 1.73 | [-0.0001, 0.001] |
