# Supplementary material for: Malleability of rumination: An exploratory model of CBT-based plasticity and long-term reduced risk for depressive relapse among youth from a pilot randomized clinical trial
Source: PLoS One. 2020 Jun 17;15(6):e0233539. doi: 10.1371/journal.pone.0233539 (PMC7299403; doi:10.1371/journal.pone.0233539)
Supplement: S5 Table — Four youth had incomplete responses on ratings either at Baseline or Week Eight due to computer/task malfunction or slow response, leaving a total of 21 youth for these analyses (AO n = 10, RFCBT n = 11). AO = assessment only; RFCBT = rumination-focused cognitive behavioral therapy. (DOCX) [file pone.0233539.s013.docx]

**S5 Table. Characteristics by treatment group for self-reported ratings of sadness and self-focus during each condition of the rumination induction task in the Manipulation Check Sample (*N*=21).**

| Condition | Treatment Group | Time | | | | | | | |
| --- | --- | --- | --- | --- | --- | --- | --- | --- | --- |
|  |  | **Baseline** | | |  | | **Week Eight** | | |
|  |  | *M (SD)* | | |  | | *M (SD)* | | |
|  |  | *Sadness* | | | | | | | |
| Rumination | **AO** | 2.77 | (0.85) | |  | | 2.87 | | (0.71) |
|  | **RFCBT** | 2.90 | (0.73) | |  | | 2.89 | | (0.65) |
| Distraction | **AO** | 1.87 | (0.71) | |  | | 2.15 | | (0.70) |
|  | **RFCBT** | 1.67 | (0.53) | |  | | 1.62 | | (0.27) |
|  |  | *Self-Focus* | | | | | | | |
| Rumination | **AO** | 3.30 | (0.42) |  | | 3.45 | | (0.51) | |
|  | **RFCBT** | 3.38 | (0.50) |  | | 3.30 | | (0.44) | |
| Distraction | **AO** | 2.55 | (0.72) |  | | 2.97 | | (0.72) | |
|  | **RFCBT** | 2.23 | (0.50) |  | | 2.13 | | (0.50) | |
