# Supplementary material for: Malleability of rumination: An exploratory model of CBT-based plasticity and long-term reduced risk for depressive relapse among youth from a pilot randomized clinical trial
Source: PLoS One. 2020 Jun 17;15(6):e0233539. doi: 10.1371/journal.pone.0233539 (PMC7299403; doi:10.1371/journal.pone.0233539)
Supplement: S6 Table — Conducted using pearson’s correlations. pDMN+ = posterior default mode and additional regions; SV-SM = salience and somatomotor network factor from [36].* p < .05, two-tailed. (DOCX) [file pone.0233539.s014.docx]

**S6 Table. Correlations between all regions different from healthy controls across Baseline and Week Eight.**

|  | **Week Eight** | | | | | | | | | | | | | | |
| --- | --- | --- | --- | --- | --- | --- | --- | --- | --- | --- | --- | --- | --- | --- | --- |
|  | **pDMN+** | | | | | | |  | **SV-SM** | | | | | | |
| **Baseline** | **1** | **2** | **3** | **4** | **5** | **6** | **7** |  | **8** | **9** | **10** | **11** | **12** | **13** | **14** |
| **pDMN+** |  |  |  |  |  |  |  |  |  |  |  |  |  |  |  |
| **1.** Lingual Occipital (-26,-70, 2) | .04 |  |  |  |  |  |  |  |  |  |  |  |  |  |  |
| **2.** Temporal Fusiform (-40, -50, -14) | .17 | .18 |  |  |  |  |  |  |  |  |  |  |  |  |  |
| **3.** Precuneus (-4, -42, 56) | .25 | .30 | .31 |  |  |  |  |  |  |  |  |  |  |  |  |
| **4.** Thalamus/Putamen/ Amygdala/PHG (12, -12, 0) | -.22 | -.03 | -.02 | -.14 |  |  |  |  |  |  |  |  |  |  |  |
| **5.**  Precuneus (24, -68, 30) | .22 | .16 | .25 | .11 | .12 |  |  |  |  |  |  |  |  |  |  |
| **6.** Superior Temporal (64, -14, 6) | .02 | .16 | .13 | .07 | .19 | .22 |  |  |  |  |  |  |  |  |  |
| **7.** Parietal Inferior (66, -34, 34) | .03 | .26 | .37 | .05 | .36 | .33 | .35 |  |  |  |  |  |  |  |  |
| **SV-SM** |  |  |  |  |  |  |  |  |  |  |  |  |  |  |  |
| **8.** Cingulate (-2, 4, 44) | .13 | .30 | .29 | .26 | .26 | .44* | .23 |  | .33 |  |  |  |  |  |  |
| **9.** Middle Temporal (-32, -72, 14) | .12 | .06 | .22 | .03 | .17 | .26 | .46* |  | .16 | .42* |  |  |  |  |  |
| **10.** Precentral (-40, -10, 44) | .09 | .27 | .21 | .06 | .28 | .34 | .20 |  | .21 | .25 | .21 |  |  |  |  |
| **11.** Lingual (32, -58, 4) | .04 | .09 | .07 | -.07 | .05 | .06 | .06 |  | .01 | .12 | .08 | .22 |  |  |  |
| **12.** Insula (40, -22, -2) | -.27 | .07 | .10 | .05 | .18 | .05 | -.02 |  | -.07 | .16 | .07 | .12 | .04 |  |  |
| **13.** Inferior Occipital (46, -72, 4) | .12 | -.004 | .07 | .003 | -.04 | .07 | .04 |  | .16 | .01 | .22 | .05 | .07 | -.16 |  |
| **14.** Precentral (56, 6, 10) | -.04 | .29 | .32 | .17 | .33 | .42* | .18 |  | .24 | .24 | .34 | .32 | .31 | .34 | .28 |
