# Supplementary material for: Malleability of rumination: An exploratory model of CBT-based plasticity and long-term reduced risk for depressive relapse among youth from a pilot randomized clinical trial
Source: PLoS One. 2020 Jun 17;15(6):e0233539. doi: 10.1371/journal.pone.0233539 (PMC7299403; doi:10.1371/journal.pone.0233539)
Supplement: S7 Table — AO = assessment only; RFCBT = rumination-focused cognitive behavioral therapy; pDMN+ = posterior default mode and additional regions; SV-SM = salience and somatomotor network factor from [36]. (DOCX) [file pone.0233539.s015.docx]

**S7 Table.** **Comparison across Baseline and Week Eight in** **activation in two neural factors during rumination versus distraction in the Quality MRI Sample and across treatment groups (*N*=25).**

|  | Baseline | |  | Week Eight | | Paired *t*-test & *p*-value |
| --- | --- | --- | --- | --- | --- | --- |
| Group | *M (SD)* | |  | *M (SD)* | |  |
|  | *pDMN+* | | | | | |
| RFCBT | 0.55 | (0.59) |  | -0.13 | (0.88) | *t*(11) = 1.97, *p* = .08 |
| AO | 0.73 | (0.92) |  | 0.05 | (0.98) | *t*(12) = 2.64, *p* = .02 |
| Total | 0.64 | (0.77) |  | -0.03 | (0.92) | *t*(24) = 3.26, *p* = .003 |
|  | *SV-SM* | | | | | |
| RFCBT | 0.56 | (0.62) |  | -0.07 | (0.89) | *t*(11) = 1.86, *p* = .09 |
| AO | 0.75 | (0.87) |  | 0.09 | (1.01) | *t*(12) = 2.76, *p* = .02 |
| Total | 0.66 | (0.75) |  | 0.01 | (0.94) | *t*(24) = 3.22, *p* = .004 |
