# Supplementary material for: Malleability of rumination: An exploratory model of CBT-based plasticity and long-term reduced risk for depressive relapse among youth from a pilot randomized clinical trial
Source: PLoS One. 2020 Jun 17;15(6):e0233539. doi: 10.1371/journal.pone.0233539 (PMC7299403; doi:10.1371/journal.pone.0233539)
Supplement: S8 Table — Categorical effect of treatment using effects coding. All other predictors represent the effect of the predictor on the average of the whole sample. SV-SM = Salience and somatomotor network Factor 1 from [36]. (DOCX) [file pone.0233539.s016.docx]

**S8 Table. Baseline activation in SV-SM during rumination induction task predicts CDRS-R depression at Week Eight, one-year, and two-year follow-up.**

|  | Week Eight | | | | One-Year | | | | Two-Year | | | |
| --- | --- | --- | --- | --- | --- | --- | --- | --- | --- | --- | --- | --- |
| Predictors | ***B* (SE)** | ***β*** | ***p*** | ***sr*** | ***B* (SE)** | ***Β*** | ***p*** | ***sr*** | ***B* (SE)** | ***β*** | ***p*** | ***sr*** |
| Intercept | 29.46 (0.53) |  | <.001 |  | 28.97 (0.53) |  | <.001 |  | 29.83 (0.53) |  | <.001 |  |
| Treatment | -7.72 (1.07) | -.84 | <.001 | -.83 | -3.31 (1.07) | -.53 | .01 | -.52 | -6.90 (1.07) | -.80 | <.001 | -.80 |
| ∆ SV-SM | -0.99 (0.61) | -.21 | .12 | -.19 | -0.99 (0.61) | -.31 | .12 | -.28 | -0.99 (0.61) | -.23 | .12 | -.20 |
| Baseline SV-SM | -2.06 (0.82) | -.33 | .02 | -.29 | -2.05 (0.82) | -.48 | .02 | -.42 | -2.05 (0.82) | -.35 | .02 | -.31 |
| Model Summary | *F* (3, 21) = 18.17, *p* < .001  Adj. *R*^2^ = .68 | | | | *F* (3, 21) = 4.76, *p* = .01  Adj. *R*^2^ = .32 | | | | *F* (3, 21) = 14.80, *p* < .001  Adj. *R*^2^ = .63 | | | |
