# Supplementary material for: Malleability of rumination: An exploratory model of CBT-based plasticity and long-term reduced risk for depressive relapse among youth from a pilot randomized clinical trial
Source: PLoS One. 2020 Jun 17;15(6):e0233539. doi: 10.1371/journal.pone.0233539 (PMC7299403; doi:10.1371/journal.pone.0233539)
Supplement: S9 Table — Hierarchical cox regressions were used to model the added benefit of SV-SM neural activation to predict relapse beyond the effect of treatment. This additional step was not significant in prediction of AMD nor MDE. AMD = any mood disorder, MDE = major depressive episode, SV-SM = salience and somatomotor network. (DOCX) [file pone.0233539.s017.docx]

**S9 Table. Activation in SV-SM does not significantly predict relapse over the following two years.**

| **Predictors** | ***B* (SE)** | ***OR*** | ***95% CI of OR*** | ***p*** |
| --- | --- | --- | --- | --- |
| *AMD Relapse* | | | | |
| Treatment | -1.12 (0.47) | 0.33 | [0.13, 0.82] | .02 |
| **First Model Summary** | *χ^2^*(1) = 6.25, *p* = .01 | | | |
| Treatment | -1.27 (0.48) | 0.28 | [0.11, 0.72] | .01 |
| ∆ SV-SM | -0.11 (0.28) | 0.90 | [0.52, 1.55] | .70 |
| Baseline SV-SM | -0.54 (0.38) | 0.59 | [0.28, 1.23] | .16 |
| **Change with Additional Variables: ∆** *χ^2^*(2) = 2.06, *p* = .36 | | | | |
| **Second Model Summary** | *χ^2^*(3) = 8.49, *p* = .04 | | | |
| *MDE Relapse* | | | | |
| Treatment | -0.91 (0.51) | 0.40 | [0.15, 1.10] | .08 |
| **First Model Summary** | *χ^2^*(1) = 3.39, *p* = .07 | | | |
| Treatment | -1.07 (0.53) | 0.34 | [0.12, 0.97] | .04 |
| ∆ SV-SM | -0.44 (0.28) | 0.64 | [0.37, 1.11] | .11 |
| Baseline SV-SM | -0.11 (0.38) | 0.90 | [0.43, 1.87] | .78 |
| **Change with Additional Variables: ∆** *χ^2^*(2) = 2.61, *p* = .27 | | | | |
| **Second Model Summary** | *χ^2^*(3) = 5.85, *p* = .12 | | | |
